# The economic impact of schistosomiasis

**DOI:** 10.1186/s40249-021-00919-z

**Published:** 2021-12-13

**Authors:** Daniele Rinaldo, Javier Perez-Saez, Penelope Vounatsou, Jürg Utzinger, Jean-Louis Arcand

**Affiliations:** 1 Department of Economics and Land, Environment, Economics and Policy Institute (LEEP), University of Exeter, Exeter, England; 2 Department of Epidemiology, Johns Hopkins Bloomberg School of Public Health, Baltimore, MD USA; 3 Swiss Tropical and Public Health Institute, Basel, Switzerland; 4 University of Basel, Basel, Switzerland; 5 Department of International Economics, The Graduate Institute of International and Development Studies, Geneva, Switzerland; 6 Fondation pour les études et recherches sur le développement international (FERDI), Clermont-Ferrand, France

**Keywords:** Schistosomiasis, Neglected Tropical Diseases, Sub-Saharan Africa, Agriculture, Water Resources Development, Poverty

## Abstract

**Background:**

The economic impact of schistosomiasis and the underlying tradeoffs between water resources development and public health concerns have yet to be quantified. Schistosomiasis exerts large health, social and financial burdens on infected individuals and households. While irrigation schemes are one of the most important policy responses designed to reduce poverty, particularly in sub-Saharan Africa, they facilitate the propagation of schistosomiasis and other diseases.

**Methods:**

We estimate the economic impact of schistosomiasis in Burkina Faso via its effect on agricultural production. We create an original dataset that combines detailed household and agricultural surveys with high-resolution geo-statistical disease maps. We develop new methods that use the densities of the intermediate host snails of schistosomiasis as instrumental variables together with panel, spatial and machine learning techniques.

**Results:**

We estimate that the elimination of schistosomiasis in Burkina Faso would increase average crop yields by around 7%, rising to 32% for high infection clusters. Keeping schistosomiasis unchecked, in turn, would correspond to a loss of gross domestic product of approximately 0.8%. We identify the disease burden as a shock to the agricultural productivity of farmers. The poorest households engaged in subsistence agriculture bear a far heavier disease burden than their wealthier counterparts, experiencing an average yield loss due to schistosomiasis of between 32 and 45%. We show that the returns to water resources development are substantially reduced once its health effects are taken into account: villages in proximity of large-scale dams suffer an average yield loss of around 20%, and this burden decreases as distance between dams and villages increases.

**Conclusions:**

This study provides a rigorous estimation of how schistosomiasis affects agricultural production and how it is both a driver and a consequence of poverty. It further quantifies the tradeoff between the economics of water infrastructures and their impact on public health. Although we focus on Burkina Faso, our approach can be applied to any country in which schistosomiasis is endemic.

**Graphical Abstract:**

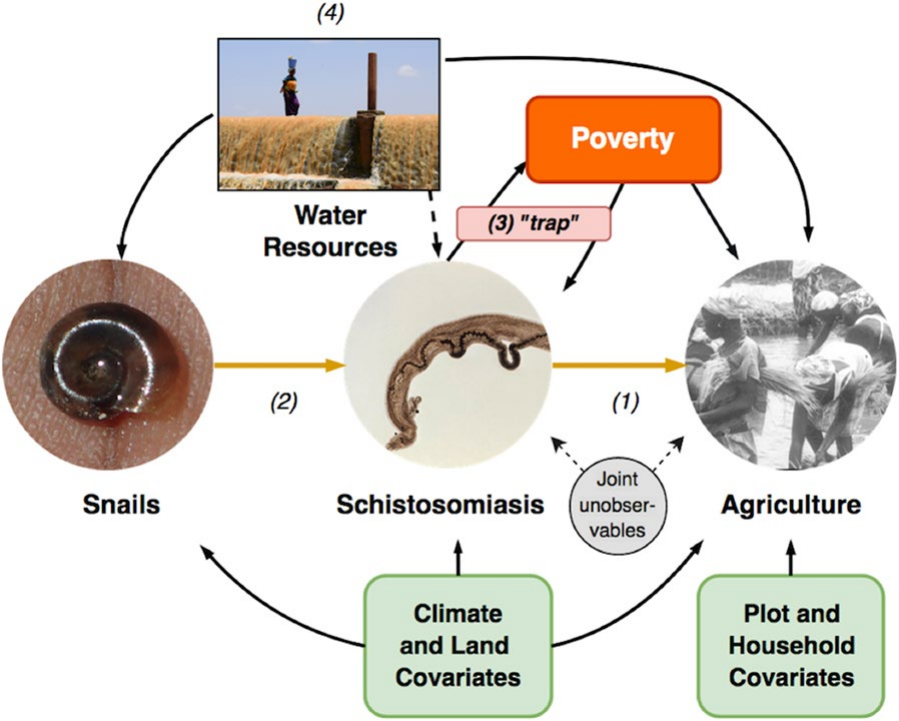

**Supplementary Information:**

The online version contains supplementary material available at 10.1186/s40249-021-00919-z.

## Background

Schistosomiasis is a water-based debilitating neglected tropical disease that affects an estimated 250 million people, more than 85% of whom live in sub-Saharan Africa [1]. Schistosomiasis claimed between 1.5 and 2.5 million disability-adjusted life years per year in the past decade [2, 3]. Severe morbidity due to schistosomiasis results from the accumulation of eggs laid by flatworms of the genus *Schistosoma* that are trapped in the tissues of the human host, leading to a chronic inflammatory response. The parasite species that cause the two main forms of the disease, intestinal (*S. mansoni*) and uro-genital (*S. haematobium*), present a complex life cycle involving two reproduction phases, the first asexual in specific freshwater snail species that act as intermediate hosts, followed by sexual in the human host. Infection occurs through skin penetration by water-motile schistosome larvae, which secrete eggs that perpetuate the parasite’s lifecycle by exiting the human host through urine or feces. A large fraction of these eggs remains trapped in the tissues surrounding the bladder or the intestine, eliciting the chronic inflammation that constitutes the root of schistosomiasis-induced morbidity [4]. When untreated, advanced forms of schistosomiasis lead to kidney failure, bladder cancer, liver fibrosis [5], as well as heightened risk of HIV transmission [6]. The highest parasite burden is usually borne by school-age children and the disease has been linked to anemia, stunting and cognitive deficits, leading to poor school performance and higher drop-out rates [7]. Due to these life-long impacts, schistosomiasis exerts large health, social and financial burdens on infected individuals and households [8].

Water resources development and management aimed at alleviating poverty in rural schistosomiasis-endemic communities in sub-Saharan Africa have been shown to exacerbate disease transmission [9]. Dams and irrigation schemes expand the suitable habitat for the freshwater snails that serve as the intermediate hosts of schistosomes, and also increase the frequency and density of human-water contacts during which infection can occur [10]. This effect is particularly marked in water-constrained regions, possibly due to the concentration of human-water contacts in the few available water points [9]. One expects in all likelihood that for low- and middle-income countries the economic sector most affected by the disease would be agriculture, particularly in its subsistence form [11, 12]. This is because populations that rely heavily on agricultural production are the ones that are the most exposed to infection and ultimately suffer the highest disease burden. However, its net economic impact and the underlying tradeoffs between water resources development and public health concerns have yet to be rigorously quantified.

**Fig. 1:**
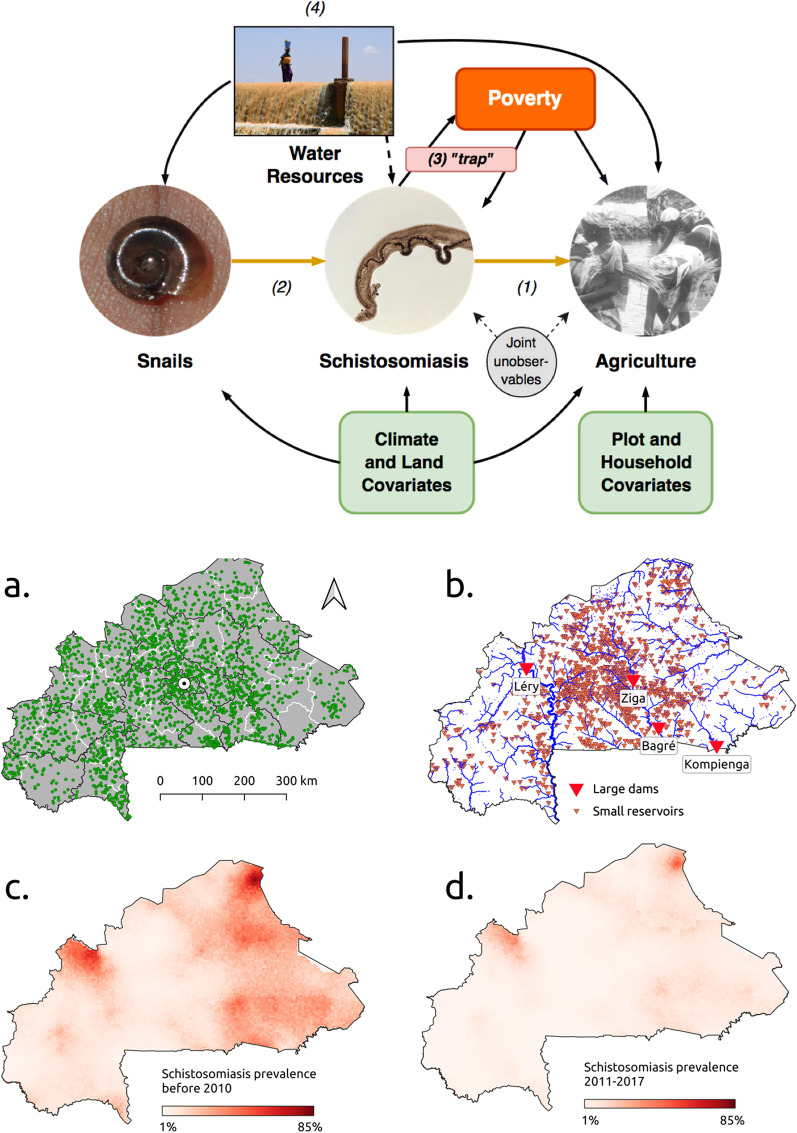
(Upper panel) Mechanisms studied in the paper. Our main interest lies in estimating (1), the causal effect of schistosomiasis on agriculture. To achieve identification we use a set of variables (instruments) that influence disease intensity without directly affecting agriculture: we use the densities of the different snail species that act as intermediate hosts for the *Schistosoma*. (2). Poverty is shown to have a reinforcing effect on the burden the disease exerts on agricultural production, as well as being its consequence (3). Water resources development (4) is shown to boost agriculture and development, but also to increase the adverse effects of schistosomiasis via both the increase of snail habitat and human-water contact. (Lower panel) Data overview. **a** Villages included in the agricultural surveys, the capital Ouagadougou (white point) and level 1 (regions, black lines) and level 2 (provinces, white lines) administrative subdivisions. **b** River network (blue lines, width proportional to upstream area) and water resources infrastructure in the country. **c** Estimated schistosomiasis prevalence up to 2010. **d** Estimated schistosomiasis prevalence for 2011–2017

We focus on Burkina Faso, a country where schistosomiasis is endemic both in its intestinal and uro-genital forms [13, 14]. Burkina Faso is a low-income, landlocked Sub-Saharan country with very limited resources. Burkinabé agriculture is the main component of the country’s economy, employing roughly 80% of the population, and is mostly of the subsistence variety, with low crop and livestock productivity and high levels of inefficiency [15, 16]. Large- and small-scale water resources development projects have been completed in the past 30 years to support agricultural activities and reduce climate vulnerability (Fig. 1b), which have however exacerbated the prevalence of malaria and schistosomiasis [17]. Mass drug administration (MDA) campaigns, initiated by the Schistosomiasis Control Initiative (SCI) in 2005 [18], have been successful in reducing morbidity in most regions with a national mean prevalence of around 5% in school-age children in 2010 (Fig. 1d) [19, 20].

Because of the complexity of the dynamics of schistosomiasis and its interlinkages with a large set of socioeconomic and environmental variables [21], identifying the relationship between the disease and economic development via its effect on productivity requires the use of detailed agricultural and household datasets, as well as precise information on disease prevalence on a large spatial scale. Recent developments in disease mapping allow us to obtain high resolution prevalence maps which have been used in a variety of public health contexts [14] but have not hitherto been paired with data on households and agricultural production. The upper panel of Fig. 1 shows a schematic representation of the mechanisms that we estimate, which are the aims of this study: the estimation of the economic impact of schistosomiasis via its effect on agricultural production, the interlinkages between the disease and poverty and the feedback effects between disease diffusion, water resources and economic development.

## Methods

### Data and context

Survey data were obtained from the National Institute of Statistics and Demography (INSD) in Ouagadougou. The annual agricultural dataset provides detailed information on crops, yields, inputs and pesticides, plot characteristics and labor type for the 2003−2017 period. Surveyed villages were distributed relatively uniformly across the country (Fig. 1a). The household survey data cover the 1996–2017 period and include detailed information on household characteristics and demographics. We first merge and synchronize both survey datasets, and geolocalize the villages using fuzzy string matching with administrative data. We include a comprehensive set of climatic remote-sensing data including precipitation, temperature, and vegetation indices such as temperature and rainfall data, land surface temperature for day and night, a normalized difference vegetation index and enhanced vegetation index. We extrapolate a variety of covariates from the raw rasters, such as mean temperatures in dry and rainy seasons, precipitations in all seasons and mean and maximum dry spells. The pixel resolution of the climate data allows us to merge the rasters with the survey datasets at the village level. Panel (a) in Fig. 1 shows the location of our study villages. We then merge the dataset with two high-resolution maps of schistosomiasis prevalence estimates in school-aged children at a pixel resolution of $5\times 5$ km. The first map applies up until 2010 (Fig. 1c), the second between 2011 and 2018 (Fig. 1d). The estimated prevalence is a joint measure of both uro-genital and intestinal schistosomiasis, and is obtained by means of Bayesian geostatistical analysis [14]. We choose the years 2009 and 2011 in order to maximize the number of repeated households (i.e. observed each year) and to provide the best fit and model diagnostics: in the Additional file 1: Appendix we show our results to be robust to the choice of different years around the 2010 cutoff. Furthermore, these years cover the sharp decrease in disease burden in school-aged children through Burkina Faso’s schistosomiasis control program observed between 2008 and 2013 [22]. Because of resolution of the disease maps, prevalence is constant within each village: households belonging to the same village will be assigned the same level of schistosomiasis prevalence. Given that health consequences of schistosomiasis are more directly linked to infection intensity (measured by egg-output) than to prevalence [23], we translate estimated prevalence into a joint measure of schistosomiasis infection intensity in terms of the average number schistosome egg-output per person (see Additional file 1: Appendix). Model-based estimates of spatial snail density were derived from malacological surveys collected in two field sites located along the South-North climatic gradient between the Sudanian and Sahelian regions [24]. Full information on the dataset, its sources, all summary statistics and details concerning the covariates used in the analysis can be found in the Additional file 1: Appendix.

### Schistosomiasis and agriculture

In order to estimate the economic impact of schistosomiasis (relationship (1) in Fig. 1), we rely on a specification that fully exploits the wealth of information available in our dataset at both plot and household levels. We identify the burden of the disease as a shock to overall productivity in an agricultural production function, which does not directly affect the amount of labor or physical inputs needed for production but instead influences their effectiveness. We begin by specifying the production technology as

1 $$\begin{aligned} Y_{ihjt} = A_{hj}(1+\phi _{hjt})^{-\theta } F(X_{ihjt}) e_{ihjt}, \end{aligned}$$

where $Y_{ihjt}$ is the yield (output per hectare) of plot *i*, farmed by household *h* in village *j* at time *t*, $\phi _{hjt}$, which is common to all plots cultivated by a given household, represents the direct effect of schistosomiasis on productivity, calibrated by a parameter $\theta$. We model the impact of the disease as $(1+\phi )^{-\theta }$ in order to represent the fact that in a disease-free state the household’s production technology is unaffected, and decreases as the disease burden worsens. $A_{jh}$ are household time-invariant productivity shifters, some of which may be unobservable, *F*(.) is the production function and $X_{ihjt}$ is the matrix of overall inputs used for agricultural production, including plot, household, climate and land covariates. Lastly, the term $\alpha ^{FE} = \{ \alpha _t + \alpha _h +\alpha _c +\alpha _{ct} + \alpha _{ht} +\alpha _{ch}\}$ allows to control for all unobservables at a household, crop and year level as well as their interactions (also known as fixed effects in the economics literature). Lastly, the term $\epsilon _{ihjt}$ as an idiosyncratic Gaussian disturbance. The parameter of interest is $\theta$: it modulates the extent to which the disease affects agricultural yield, and we expect its estimate to be negative. Since the “real” effect $\phi$ of the disease is unobservable, we proxy it with the measure of infection intensity in terms of mean egg-output per person discussed earlier, denoted by $I_{jt} = (1+Int_{jt})$. Taking logarithms then results in the quasilinear model

2 $$\begin{aligned} y_{ihjt} = {\tilde{A}}_{jh} - \theta {\tilde{I}}_{jt} + {\tilde{F}}(X_{ihjt}) + \alpha ^{FE} + \epsilon _{ihjt}, \end{aligned}$$

where our goal is to identify the parameter $\theta$. We estimate this burden first by means of multiplicative (Cobb-Douglas) form for the production technology. We then refine this estimate by absorbing all the non-linear confounding effects stemming from the large matrix of inputs by means of various machine learning methods, compared with a mean squared error criterion. In order to check for nonlinearities in the burden, we fit an instrumented adaptive spline for the schistosomiasis intensity variable using a two-stage semiparametric method on the log-linear model. In the Additional file 1: Appendix we report all details concerning the estimation framework and show how this interpretation of schistosomiasis as a productivity shock is not rejected by the data, and is compatible with a household optimization model. Since the “real” effect of the disease on household members is unobservable, we approximate it by the measure of infection intensity in terms of mean egg-output per person, as discussed earlier. This implies that plot-level estimation is likely to be affected by biases stemming from endogeneity issues, and we address these with the use of an instrumental variables (IV) strategy. This requires the choice of a set of variables (instruments) that are strongly related to the presence of schistosomiasis, and affect agricultural yield only via the disease burden. A natural choice is constituted by the densities of the snails that act as intermediate hosts of the schistosomes. The presence of either kind of aquatic snail is directly linked to the prevalence of both forms of schistosomiasis, whilst not having any direct detrimental effect on agricultural yields. In order to control for any effects that might jointly determine the presence of snails and agricultural production, we include a plethora of covariates that account for climate, precipitations and land characteristics, and use the snail predictions lagged by one year in order to avoid simultaneous dependance on joint unobservables. Furthermore, snail control strategies are shown to have potential benefits for morbidity control [25, 26], validating the theoretical relevance of our IVs. Snail densities are thus likely to constitute ideal instruments, and allow us to estimate the causal effect of interest. This corresponds to relationship (2) in the upper panel of Fig. 1. In the Additional file 1: Appendix we report a detailed justification of the validity of this specification, as well as the complete description of the models, the covariates and the estimation methods as well as all additional tables, results and robustness checks.

### Schistosomiasis and poverty

In Burkina Faso, agricultural households are largely engaged in subsistence farming: shocks to yield therefore affect simultanously both income and survival probability. Moreover, it is likely that the productivity decrease attributable to schistosomiasis is a function of various household characteristics. To account for this, we examine the additional burden of the disease for households farming plots that belong to different quantiles of the joint distribution of plot surface and crop weight. We interact schistosomiasis intensity with an indicator representing whether a given plot belongs to a specific quantile of the joint plot surface/crop weight distribution. The estimated equation is the following:

3 $$\begin{aligned} y_{ihjt}= & {} {\tilde{A}}_{jh} + \theta {\tilde{I}}_{jt} + \theta _{pj} {\tilde{I}}_{jt}\times \mathbbm {1}_{ ( w_j, s_j) \le Q^j_k} + {\tilde{F}}(X_{ihjt}) + \alpha ^{FE} + \epsilon _{ihjt} \nonumber \\ Q^j_k= & {} \min \{w_{ihjt}, \text {s}_{ihjt} : k \le \Phi (w, \text {surf}; t) \}, \end{aligned}$$

where $\Phi$ is the joint distribution of plot weight *w* and surface *s* for each year *t*. The coefficient of interest is $\theta _{pj}$, estimated varying the indicator *k* on a grid ranging from the 20th to the 70th percentile of the joint distribution.

In order to characterize this mechanism in terms of its link to household poverty, we then carry out a similar procedure where the indicator function is defined at the household rather than at the plot level. The cutoffs in this case correspond to the lower 5% and 10% tails of the joint distribution of total harvest weight and plot surface farmed by each household. The interest is in the estimation of $\theta _{ph}$ in

4 $$\begin{aligned} y_{ihjt}= & {} {\tilde{A}}_{jh} + \theta {\tilde{I}}_{jt} + \theta _{ph} {\tilde{I}}_{jt} \times \mathbbm {1}_{(w^h,s^h) \le Q^h_k} + {\tilde{F}}(X_{ihjt}) +\alpha ^{FE} + \epsilon _{ihjt} \nonumber \\ Q^h_k= & {} \min \left\{ \sum _{j=1}^{N^h_{t}} w_{ihjt}, \sum _{j=1}^{N^h_{t} } \text {s}_{ihjt} : k < \Phi \left( w^h, \text {s}^h; t \right) \right\} , \end{aligned}$$

where $\Phi \left( w^h, \text {s}^h \right)$ is the joint distribution of total crop weight and plot surface farmed by each household and $N^h_{t}$ is the number of plots farmed by each household each year.

### Schistosomiasis and water resources development

Given that schistosomiasis is water-based, we study the feedback effects between the disease and water resources development. We begin by concentrating our attention on Burkina Faso’s four main dams, shown in (Fig. 1b). We aggregate the data up to the village level, and we compute the geographical distance of each village from the closest dams and reservoirs. By interacting the presence of a dam with our measure of disease intensity, we disentangle the direct impact of dams, which should increase yields, from the deleterious indirect effects that they may produce by facilitating the diffusion of schistosomiasis. For this estimation we rely on an instrumented spatial autoregressive specification (SAR). The estimated equation is

5 $$\begin{aligned} y_{jt} = (1_{2j} - \rho W_j)^{-1}[ \theta _1 I_{jt} + \theta _{2} \mathbbm {1}_{dam} + \theta _{int} I_{jt} \times \mathbbm {1}_{dam}+ {\tilde{X}}_{jt}\beta + \alpha _t + \alpha _r + \epsilon _{jt}], \end{aligned}$$

where $\alpha _{t,r}$ accounts for time and region unobservables, $W_j$ is a matrix of spatial weights given by the between-villages distance in coordinate degrees and $\rho$ is the spatial correlation parameter, fit by maximum likelihood.

We refine the previous results by accounting for each village’s distance in km from the nearest dam or water reservoir. The full network of dams and reservoirs is illustrated in panel (b) of Fig. 1. We interact the presence of a large dam with the distance from any water infrastructure as well as with the measure of intensity. The estimated equation is

6 $$\begin{aligned} y_{jt}\,= & {} (1_{2j} - \rho W_j)^{-1}[ \theta _1 I_{jt} + \theta _2 dist_{j} + \theta _3 \mathbbm {1}_{dam} + \theta _{3int}I_{jt} \times dist_j \times \mathbbm {1}_{dam} + \nonumber \\&+ \theta _{2int1} I_{jt} \times dist_j + \theta _{2int2} I_{jt} \times \mathbbm {1}_{dam} + \theta _{2int3} dist_{j} \times \mathbbm {1}_{dam}+ \nonumber \\&+{\tilde{X}}_{jt}\beta + \alpha _t+ \alpha _r + \epsilon _{jt}]. \end{aligned}$$

where the main coefficients of interest are $\theta _{3int}$ and $\theta _{2int1}$. All details and robustness checks are again reported in the Additional file 1: Appendix.

### Data analysis

All estimations have been obtained using the software R 3.6.2 (https://cran.r-project.org/). The dataset as well as the main variables of interest are described in full in the Additional file 1: Appendix. Inference on the estimated coefficients has been done by means of cluster-bootstrapping standard errors at the village level in order to match the resolution of the disease and climate maps.

## Results

### Schistosomiasis and agriculture

**Fig. 2:**
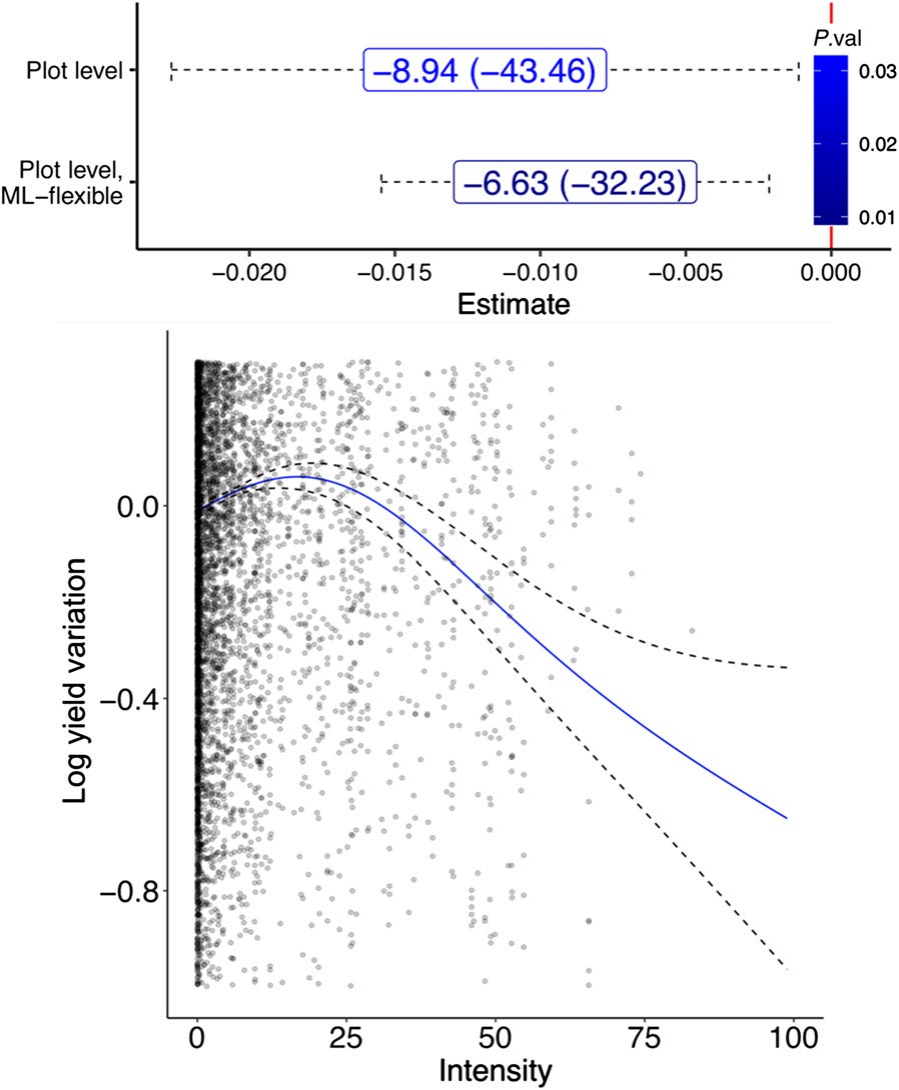
(Upper panel) Estimates of the yield loss (in %) due to schistosomiasis. Each label reports the average loss and in parentheses the loss at the top 5% infection intensity clusters. 95% error bands are cluster-bootstrapped at the village level. (Lower panel) Nonlinear effect of the disease intensity. Dotted lines represent the 95% confidence interval in function fitting

In Fig. 2 we present results for households and plots observed in 2009 and 2011. All results use our snail density measures as IVs for schistosomiasis intensity. The upper panel of Fig. 2 reports the main estimates of the paper: the estimates for the parameter $\theta$ in Eq. (2) can be found on the *x*-axis of the Figure, which quantify the loss of agricultural yield due to schistosomiasis. The intensity measure is expressed in terms of worm eggs per person, and is a joint measure of eggs per gram of stool (*S. mansoni*) and eggs per 10 ml of urine (*S. haematobium*). The coefficient is expected to be negative and in the order of $10^{-2}$-$10^{-3}.$ The point estimate represents the marginal percentage impact of one additional worm egg per person on yield. In order to provide a more intuitive measure of the economic impact of schistosomiasis, in the labels we present the percentage loss of agricultural yield due to schistosomiasis suffered by the households that experience the average infection intensity. In parenthesis we report the agricultural loss suffered by the households at the top 5% infection intensity clusters. Standard errors are cluster-bootstrapped at a village level, matching the level of measurement at which the disease intensity is measured.

The topmost estimate in the upper panel of Fig. 2 results from the log-linear specification. The point estimate of -0.0119 (s.e. 0.0057, *P*=0.035) shows that schistosomiasis causes an average loss of agricultural yield of 8.9% [95% confidence interval (*CI*): 1.1−16.5%], rising to 43.5% for the households in the top 5% quantile of disease intensity. The second estimate is obtained via adaptive methods for the production technology: by doing so, point estimates of the the disease intensity’s marginal effect fall, and precision improves. For our 2009−2011 sample, our preferred method is given by tuned random forests, and results in an estimate of -0.0088 (s.e. 0.0035, *P *= 0.013). This estimate of the marginal effect of disease intensity on log yield implies a mean loss of agricultural yield caused by schistosomiasis of 6.6% (95% *CI*: 2.2−12%), increasing to 32.2% for the household at the top 5% infection quantile. All plot-level estimates control for time, household and crop unobservables as well as their interactions. Non-linearities in the impact of schistosomiasis are significant, as illustrated in the lower panel of Fig. 2. The adverse effect of the disease is concentrated in villages that experience mid- to high levels of disease intensity, with the negative effect on yield starting to become significant at intensities above 30 worm eggs/person.

### Schistosomiasis and poverty

**Fig. 3:**
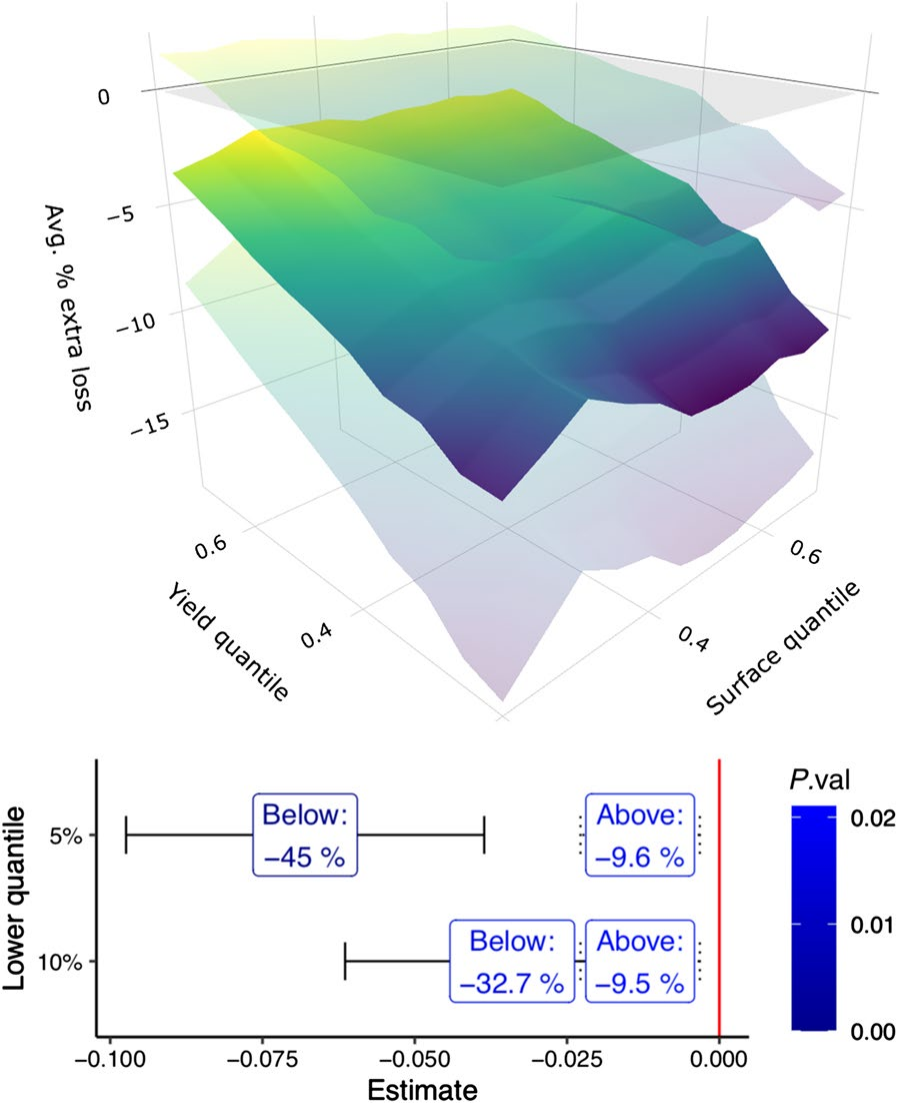
(Upper panel) Added schistosomiasis-induced yield loss due to poverty. Each point on the middle surface represents the extra loss due to the disease for plots below the respective crop weight and surface quantiles. The upper and lower transparent surfaces are cluster-bootstrapped 95% confidence intervals. (Lower panel) Losses to yield suffered by households above and below threshold levels of poverty, defined by the left tail of the joint harvest weight and plot surface distribution

Figure 3 reports the results concerning the interaction between schistosomiasis and poverty. The upper panel presents the estimates of $\theta _{pj}$ in Eq. (3) for each grid point of the joint distribution of plot-level harvest weight and plot surface. The results show that plots that belong to the lowest tail of joint production and surface suffer an additional loss to yield due to schistosomiasis ranging from 5% to 10%. These incremental effects stop being significant at the 55th percentile, and the full set of estimates is presented in the Additional file 1: Appendix. The lower panel presents the estimates of $\theta _{pj}$ in Eq. (4). This set of results explores the burden stemming from the presence of household poverty, estimating the additional yield loss due to schistosomiasis suffered by households that farm plots at the lowest quantiles of the joint plot surface and harvest weight distribution. We find that for such households schistosomiasis exerts a disproportionately higher burden: −32.7% (95% *CI*: 22.2−43.2%, estimate: −0.034, s.e. 0.014, *P *= $7$ $\times$ $10^{-3}$) for the households in the bottom 10% and -45% (95% *CI*: 33.1−56.9%, estimate: −0.068, s.e. 0.015, *P *= 0.01) for those in the bottom 5%.

### Schistosomiasis and water resources development

**Fig. 4:**
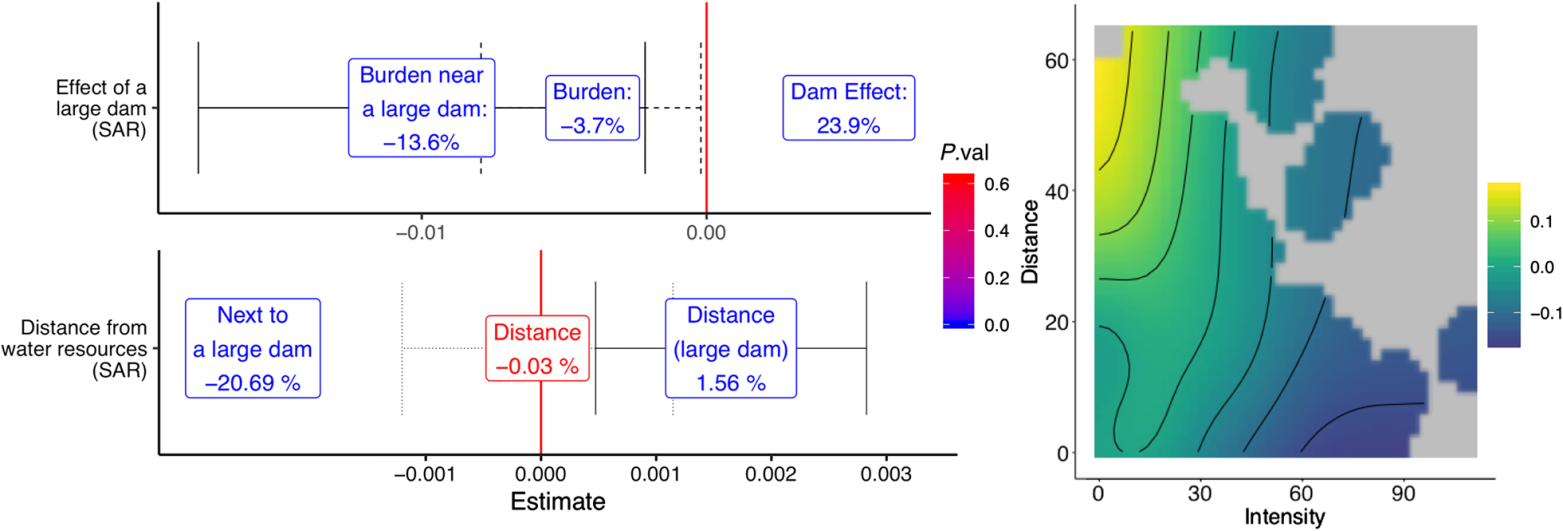
(Top left) Added effect of schistosomiasis on yields caused by the presence of a large dam. (Bottom left) Joint effect of schistosomiasis, distance from water resources networks and dam size. Estimations account for spatial correlation. (Right) Joint effect of schistosomiasis and distance (in km) from dams and water networks. Each point in the fitted surface represents the effect of schistosomiasis on yield for a village at the corresponding distance from a dam or a water reservoir: the darker the color, the more negative is the effect

Figure 4 reports the findings concerning the feedback effects of water resources development and disease diffusion. The explicit inclusion of the presence of a dam induces spatial effects in the data that do not vanish even after controlling for a larger-scale set of unobservables, contrary to our earlier results. This is confirmed by how the Moran’s *I* statistic for the village-level model, even after controlling for region-specific effects, is still significantly positive (0.10, highly significant). Indeed, it is only when a SAR framework is adopted that the separate effects of intensity and dams are estimated with sufficient precision to make them statistically significant. The full set of results and robustness checks can be found in the Additional file 1: Appendix. The top left panel of Fig. 4 presents the results of the estimation of $\theta _1, \theta _2$ and $\theta _{int}$ in (5). These results show that whilst the presence of a dam ($\theta _2$)increases yield by 23.9% (95% *CI*: 21.7−26.2%), the average burden due to schistosomiasis for villages within a radius of 30 km around each of the five large-scale dams of Burkina Faso ($\theta _{int}$) is a yield loss of 13.6% (95% *CI*: 2.3−25.2%. Estimate: −0.01, s.e. 0.0036, *P *= 0.004). Villages outside this radius do not benefit significantly from the direct effect of the dams, but incur average losses due to the disease ($\theta _1$) of only 3.7% (95% *CI*: 2.9–4.5%. Estimate: −0.004, s.e. 0.002, *P* = 0.01). The bottom left panel of Fig. 4 presents the results that separate the distance effect between large dams and smaller scale infrastructures, represented by Eq. (6). For villages located within 1 km from a large dam, the average estimated loss due to schistosomiasis is 20.7% ($\theta _{2int2}$: estimate -0.021, s.e. 0.005, *P*=1.3$\times$ $10^{-5}$). An increase of 1 km in distance from the dam generates an average 1.5% *reduction* of the additional burden of schistosomiasis on agricultural yield ($\theta _{3int}$: estimate 0.0013, s.e. 0.0005, *P *= 0.005): being further away from large dams is therefore beneficial. The distance effect for villages *not* in proximity of large dams ($\theta _{2int1}$), however, is insignificant at any level of confidence (*P *= 0.46): such dams seem indeed to be the main culprits of the feedback effects. The right-hand panel of Fig. 4 shows how the deleterious marginal effect of schistosomiasis intensity on yield is mitigated as one moves further away from a dam or a reservoir, as well as how areas with high schistosomiasis intensity are concentrated within 20−30 km of a dam or reservoir. Aggregating at the village level also allows us to study the impact of spatial correlation, with the key issue being whether controlling for higher (commune) level unobservables is sufficient to control for spatial correlation. We begin by estimating Moran’s *I* for the models with only time fixed effects as opposed to time and commune fixed effects: spatial correlation falls from 0.13 - low, but statistically significant at all conventional levels of confidence (*P *= 2$\times 10^{-5}$) to a statistically insignificant -0.07 (*P *= 0.970). Commune level unobservables therefore seem to lie at the root of any spatial correlation present in the data.

## Discussion

Beyond general results concerning the relationship between diseases and economic development [27–30], there is a relatively limited amount of research that focuses on the economic impact of endemic diseases. This is particularly true of parasitic diseases such as schistosomiasis. Attempts have been sparse and either focused on specific mechanisms of small scale, albeit of great importance, or lacked results of significant strength. St. Lucia was one of the first countries studied in an effort to ascertain the impact of schistosomiasis on labour productivity and agricultural production [31–33]: very little was detected, but mismeasured variables and lack of data hindered the effort. A quasi-experiment carried out in Mali found no direct effects on rice production, but did find effects of the disease incidence on the use of labour and other resources within households [34]. Conversely, negative effects of schistosomiasis on rice production have been detected for infected households in the Cameroon [35]. A deworming project in Kenya that included schistosomiasis, in which school-based mass treatment with deworming drugs were randomly allocated in schools, obtained evidence of large health benefits and increased school participation, although without establishing significant treatment effects on academic performance [36].

In this paper we have studied the impact of schistosomiasis on agricultural yields in Burkina Faso by characterizing it as an adverse shock to the productivity of farming households. By merging rich agricultural and household datasets with high-resolution disease maps, we created a combined dataset which is one of the major contributions of this paper. We have shown how schistosomiasis has a large, negative and nonlinear effect on agricultural productivity. All estimates include a measure of malaria prevalence in order to account for co-morbidity which, interestingly, does not significantly affect yield at conventional levels of confidence. In no way does this imply that malaria has no adverse effect on households: this simply reflects the fact that malaria exerts a burden of an entirely different nature and more compatible with a shock to labor supply [37, 38]. Losses attributable to the disease range from a mean value of 6.6% to 32% for households and villages located in areas in the top 5% infection clusters: furthermore, such losses are concentrated on the areas that suffer the upper two terciles of disease intensity. Given that grain output represents roughly 12% of Burkina Faso’s gross domestic product (GDP), our preferred mean estimate implies that schistosomiasis is associated with economic losses which correspond to around 0.8% of GDP. The upshot from the policy perspective is that the adverse impact of schistosomiasis on economic development is substantial, and control efforts aiming at reducing disease morbidity would produce substantial gains in agricultural productivity. The non-linearity of the economic burden of schistosomiasis, as shown in the lower panel of Fig. 2, further highlights the potential benefits of morbidity and transmission control in the areas where infection is highly endemic.

Having identified the nature of the economic burden of schistosomiasis as a productivity shock, we then study the role of two of its potential drivers: cropping patterns and poverty. We first focus on plots that produce the main cash crop farmed by Burkinabé households, cotton. In the Additional file 1: Appendix we show that such plots are significantly larger and seem to avoid any significant deleterious effects of schistosomiasis. A potential reason for this could be that households farming cash crops such as cotton on large plots are on average much richer: they are therefore more likely to have readier access to clean running water, enjoy better sanitary conditions and have an improved access to healthcare, including the antischistosomial drug praziquantel. In contrast, households that rely on food crops for subsistence agriculture farm smaller plots and suffer the majority of the burden. However, cash crops, particularly genetically modified (GMO) insect-resistant varieties of cotton, are also farmed by poorer households in order to reduce the risk of adverse shocks. For this reason, we focus on the added burden of schistosomiasis linked with the underlying plot characteristics such as surface and output per hectare, which are likely to be correlated with poverty at the household level. Fig. 3 shows how smaller and less productive plots tend to suffer a higher burden, and how this phenomenon is further amplified for households that farm plots that jointly have the smallest surface and yield. Households in the lower reaches of this distribution correspond to those who are the most affected by poverty, almost entirely dependent on subsistence agriculture, and we see how such households experience a disproportionately higher burden than do richer ones. Our findings thus provide evidence that schistosomiasis is effectively a disease of poverty, acting both as its cause and consequence, as indicated in relationship (3) in Fig. 1. Poverty strongly reinforces the negative economic impact of schistosomiasis, with this feedback loop potentially generating a “poverty trap” phenomenon in which the debilitating nature of the disease locks households in a vicious cycle of lower agricultural productivity and consequent greater indigence. Conversely, development interventions that increase agricultural productivity and allow peasants to diversify into cash crops will both improve living standards and reduce the economic burden of the disease.

Having established the reinforcing effects of schistosomiasis on poverty, it is of considerable importance to examine whether economic policies aimed at lifting people out of poverty can indirectly increase poverty itself by means of their impact on the spatial distribution of the disease, as indicated by mechanism (4) in Fig. 1. A recent hypothesis on the origin of this mechanism has been made on the dams affecting the reproduction of the river prawns feeding on the snail hosts [39]. Our results indicate that while Burkina Faso’s main dams, all else constant, increase agricultural yields, they can also induce substantial negative feedback effects upon the economic burden of schistosomiasis, roughly doubling it for villages located within a 30 km radius. It is therefore important from a global health perspective to investigate whether it is only large-scale dams that are the culprits when it comes to the transmission of the disease, or whether smaller scale projects built for livestock and irrigation can potentially generate as much of an adverse effect. We find that households located in areas surrounding Burkina Faso’s four main dams suffer from large negative feedback effects between schistosomiasis and water resources development, whilst this effect is not significant for smaller-scale infrastructures. This implies that real returns on large-scale water resources are substantially reduced. The consequences of this result from the standpoint of poverty and inequality can be substantial: populations that gain the most from such large irrigation projects often do not correspond to those most exposed to their deleterious consequences in terms of health, productivity and general well-being. Such large-scale infrastructures account for a significant portion of development finance [40], and thus smaller-scale *in loco* water networks and reservoirs should be incentivized.

The potential limitations of this study lie mostly in two aspects: the first is the exclusive focus on Burkina Faso, thus restricting the generalizability of our results to a wider range of countries and populations. The second is the absence in our analysis of the aspects of human mobility and migration, both of which are important factors in the transmission of schistosomiasis. The first aspect, which we believe to be more of a benchmark than a limitation, is discussed in the conclusions. The second aspect, however, still remains an exciting open question which we leave to future research, particularly aimed towards the creation of a theoretical framework that includes the decision-making process of the affected populations coupled with the spatial dynamics of the disease.

## Conclusions

Economic systems are vulnerable to widespread and persistent diseases, particularly in low- and middle-income countries. Despite extensive evidence concerning the long-term health effects of parasitic diseases in developing countries, so far there have been few attempts to quantify their economic impact. There is an extensive literature on both the epidemiology and the ecology of schistosomiasis which shows how its diffusion patterns are closely related to anthropic factors. Another substantial literature studied the feedback between water resources development and disease prevalence and considers the disease to be both labor-impairing and poverty-reinforcing. Furthermore, recent developments in schistosomiasis mapping allow one to obtain reliable prevalence measures at a small geographical scale, which can be used to exploit the wealth of disaggregated socioeconomic characteristics in the estimation of the human cost of the disease. We bridge the gap between the epidemiological, ecological and economic literature: we present rigorous estimates of the economic impact of schistosomiasis, obtained by creating a new dataset that joins detailed agricultural and household survey datasets with high-resolution disease maps, and by using new methods. We show how schistosomiasis imposes a large adverse economic burden, causing an average 6.6% loss of agricultural production, a loss that increases to 32% for households in high-intensity disease clusters. We therefore estimate intervention and control efforts to have a potential impact of fundamental importance in alleviating both the economic and health consequences of schistosomiasis. We show the disease to be both a cause and a consequence of poverty and obtain a measure of returns to water infrastructures that considers their broader effects on public health.

This study focuses exclusively on Burkina Faso, which could be seen as a limitation of its scope. The reason behind this particular choice is in part because the country is likely to be a worst-case scenario due to the country’s joint economic and epidemiological profile. However, similar conditions apply to many other countries in the sub-Saharan region, where agriculture is the main economic activity and is mostly of the subsistence type, and schistosomiasis thus affects both livelihood and survival probability of the affected population. Our work highlights how the study of the interactions between disease diffusion and economic development can benefit from the use of high-resolution data, which allows one to control for the numerous confounding factors that data at higher levels of aggregation necessarily miss. Extending our framework to other regions would therefore require a data resolution at least equivalent to the one used in this paper. Conditional on this availability, our approach is general and can be applied to any other country in which schistosomiasis is endemic and indeed, to the economic impact of many other diseases.

## Supplementary Information

**Additional file 1:** Details concerning the dataset, the mathematical framework, the estimation methods and the full set of results and robustness checks.

## Data Availability

For replication purposes we release the data and code used in the paper at https://github.com/daniele-rinaldo/economic_impact_schisto.

## Abbreviations

MDA: Mass drug administration
SCI: Schistosomiasis control initiative
INSD: Institut National de la Statistique et de la Démographie (National Institute for Statistics and Demographics), Ouagadougou, Burkina Faso
IV: Instrumental variables
SAR: Spatial autoregressive models
GDP: Gross domestic product
GMO: Genetically modified organisms.

## References

[1] Walz Y, Wegmann M, Dech S, Vounatsou P, Poda J-N, NGoran E (2015). Modeling and validation of environmental suitability for schistosomiasis transmission using remote sensing. PLoS Neglect Trop D..

[2] King CH, Galvani AP (2018). Underestimation of the global burden of schistosomiasis. Lancet.

[3] James SL, Abate D, Abate KH, Abay SM, Abbafati C, Abbasi N (2018). Global, regional, and national incidence, prevalence, and years lived with disability for 354 diseases and injuries for 195 countries and territories, 1990–2017: a systematic analysis for the global burden of disease study 2017. Lancet.

[4] Colley DG, Bustinduy AL, Secor WE, King CH (2014). Human schistosomiasis. Lancet.

[5] Richter J (2003). The impact of chemotherapy on morbidity due to schistosomiasis. Acta Trop..

[6] Mbabazi PS, Andan O, Fitzgerald DW, Chitsulo L, Engels D, Downs JA (2011). Examining the relationship between urogenital schistosomiasis and HIV infection. PLoS Neglect Trop Dis..

[7] Ezeamama AE, Bustinduy AL, Nkwata AK, Martinez L, Pabalan N (2018). Cognitive deficits and educational loss in children with schistosome infection? A systematic review and meta-analysis. PLoS Neglect Trop Dis..

[8] King CH (2010). Parasites and poverty: the case of schistosomiasis. Acta Trop..

[9] Steinmann P, Keiser J, Bos R, Tanner M, Utzinger J (2006). Schistosomiasis and water resources development: systematic review, meta-analysis, and estimates of people at risk. Lancet Infect Dis..

[10] Diakité NR, Winkler MS, Coulibaly JT, Guindo-Coulibaly N, Utzinger J, N’Goran EK (2017). Dynamics of freshwater snails and Schistosoma infection prevalence in schoolchildren during the construction and operation of a multipurpose dam in central Côte d’Ivoire. Infect Dis Pov..

[11] De Janvry A, Sadoulet E (2010). Agricultural growth and poverty reduction: additional evidence. World Bank Res Obser..

[12] Christiaensen L, Demery L, Kuhl J (2011). The (evolving) role of agriculture in poverty reduction? An empirical perspective. J Dev Econ..

[13] Poda J, Traoré A, Sondo BK (2004). L’endémie bilharzienne au Burkina Faso. Soc Pat Exot..

[14] Lai Y-S, Biedermann P, Ekpo UF, Garba A, Mathieu E, Midzi N (2015). Spatial distribution of schistosomiasis and treatment needs in Sub-Saharan Africa: a systematic review and geostatistical analysis. Lancet Infect Dis..

[15] World Bank Results: Agriculture as a Powerful Instrument for Poverty Reduction (2017). http://www.worldbank.org/en/results/2017/06/29/burkina-faso-agriculture-as-a-powerful-instrument-for poverty-reduction. Accessed 16 May 2020.

[16] Udry C (1996). Gender, agricultural production, and the theory of the household. J Polit Econ..

[17] Boelee E, Cecchi P, Koné A (2010). Health impacts of small reservoirs in Burkina Faso.

[18] Garba A, Touré S, Dembelé R, Bosque-Oliva E, Fenwick A (2006). Implementation of national schistosomiasis control programmes in West Africa. Trends Parasitol..

[19] Lo NC, Lai Y-S, Karagiannis-Voules D-A, Bogoch II, Coulibaly JT, Bendavid E (2016). Assessment of global guidelines for preventive chemotherapy against schistosomiasis and soil-transmitted helminthiasis: a cost-effectiveness modelling study. Lancet Infect Dis..

[20] Coulibaly JT, Panic G, Silué KD, Kovač J, Hattendorf J, Keiser J (2017). Efficacy and safety of praziquantel in preschool-aged and school-aged children infected with *Schistosoma mansoni*: a randomised controlled, parallel-group, dose-ranging, phase 2 trial. Lancet Glob Health.

[21] Gurarie D, Seto EY (2009). Connectivity sustains disease transmission in environments with low potential for endemicity: modelling schistosomiasis with hydrologic and social connectivities. J Roy Soc Interface.

[22] Ouedraogo H, Drabo F, Zongo D, Bagayan M, Bamba I, Pima T (2016). Schistosomiasis in school-age children in Burkina Faso after a decade of preventive chemotherapy. B. World Health Organ..

[23] King CH, Dangerfield-Cha M (2008). The unacknowledged impact of chronic schistosomiasis. Chron Ill..

[24] Perez-Saez J, Mande T, Ceperley N, Bertuzzo E, Mari L (2016). Hydrology and density feedbacks control the ecology of intermediate hosts of schistosomiasis across habitats in seasonal climates. Proc Natl Acad Sci..

[25] Li EY, Gurarie D, Lo NC, Zhu X, King CH (2019). Improving public health control of schistosomiasis with a modified who strategy: a model-based comparison study. Lancet Glob Health.

[26] Knopp S, Person B, Ame SM, Ali SM, Hattendorf J, Juma S (2019). Evaluation of integrated interventions layered on mass drug administration for urogenital schistosomiasis elimination: a cluster-randomised trial. Lancet Glob Health.

[27] Acemoglu D, Johnson S (2007). Disease and development: the effect of life expectancy on economic growth. J Pol Econ.

[28] Bloom DE, Canning D, Sevilla J (2004). The effect of health on economic growth: a production function approach. World Dev..

[29] Bleakley H, Lange F (2009). Chronic disease burden and the interaction of education, fertility, and growth. Rev Econ Stat..

[30] Audibert M (2010). Endemic diseases and agricultural productivity: challenges and policy. J Afr Econ..

[31] Foster R (1967). Schistosomiasis on an irrigated estate in east Africa. III. effects of asymptomatic infection on health and industrial efficiency. J Trop Med Hyg..

[32] Baldwin RE, Weisbrod BA (1974). Disease and labor productivity. Econ Dev Cult Change.

[33] Weisbrod BA, Andreano RL, Baldwin RE, Epstein EH, Kelley AC, Helminiak TW (1974). Disease and economic development: The impact of parasitic diseases in St. Lucia Int J Soc Econ.

[34] Audibert M, Etard J-F (1998). Impact of Schistosomiasis on Rice Output and Farm Inputs in Mali. J Afr Econ..

[35] Audibert M (1986). Agricultural non-wage production and health status: a case study in a tropical environment. J Dev Econ.

[36] Miguel E, Kremer M (2004). Worms: identifying impacts on education and health in the presence of treatment externalities. Econometrica.

[37] Asenso-Okyere K, Asante FA, Tarekegn J, Andam KS (2011). A review of the economic impact of malaria in agricultural development. Ag Econ..

[38] Audibert M, Mathonnat J, Henry M (2003). Malaria and property accumulation in rice production systems in the savannah zone of cote d’ivoire. Trop Med Int Health.

[39] Sokolow SH, Jones IJ, Jocque M, La D, Cords O, Knight A (2017). Water, dams, and prawns: novel ecological solutions for the control and elimination of schistosomiasis. Lancet.

[40] Szabó S, Moner-Girona M, Kougias I, Bailis R, Bódis K (2016). Identification of advantageous electricity generation options in sub-Saharan Africa integrating existing resources. Nat Energy.

