# Supplementary material for: The economic impact of schistosomiasis
Source: Infect Dis Poverty. 2021 Dec 13;10:134. doi: 10.1186/s40249-021-00919-z (PMC8667389; doi:10.1186/s40249-021-00919-z)
Supplement: Supplementary file 1 — Additional file 1: Details concerning the dataset, the mathematical framework, the estimation methods and the full set of results and robustness checks. [file 40249_2021_919_MOESM1_ESM.pdf]

The economic impact of schistosomiasis
Appendix

Daniele Rinaldo[1a], Javier Perez-Saez[b], Penelope Vounatsou[c,d], Jürg Utzinger[c,d], Jean-Louis Arcand[e,f]

[a] *Department of Economics and Land, Environment, Economics and Policy Institute (LEEP), University of Exeter, United Kingdom*
[b] *Department of Epidemiology, Johns Hopkins Bloomberg School of Public Health, Baltimore, USA*
[c] *Swiss Tropical and Public Health Institute, Basel, Switzerland*
[d] *University of Basel, Switzerland*
[e] *Department of International Economics, The Graduate Institute of International and Development Studies, Geneva, Switzerland*
[f] *Foundation for Studies and Research on International Development (FERDI), Clermont-Ferrand, France*

Contents

1 Data **1**
1.1 Agricultural and Household data . . . 1
1.2 Climatic, environmental and malaria covariates . . . 3
1.3 Transforming schistosomiasis prevalence into infection intensity . . . 3

2 Methods **6**
2.1 Framework and estimation methods . . . 6
2.1.1 Schistosomiasis and agriculture . . . 7
2.1.2 Schistosomiasis and poverty . . . 9
2.1.3 Schistosomiasis and water resources development . . . 9
2.2 Schistosomiasis and optimal input allocation . . . 10
2.3 Snail densities as instrumental variables . . . 11

3 Schistosomiasis and agriculture **14**
3.1 Full results . . . 14
3.2 Non-linearity and interactions . . . 17
3.3 Other determinants of agriculture in the instrumented linear regressions . . . 19

4 Schistosomiasis and poverty: subsistence farming, crop choices and burden heterogeneity **19**

5 Schistosomiasis and water resources development: full results **24**

1. Data

1.1. Agricultural and Household data

The agricultural and household surveys we use has been obtained from the Direction Générale des Previsions et de Statistiques Agricoles at the Institut National de la Statistique et la Démographie in Ouagadougou

[1]Corresponding Author.
Email address: d.rinaldo@exeter.ac.uk (Daniele Rinaldo)

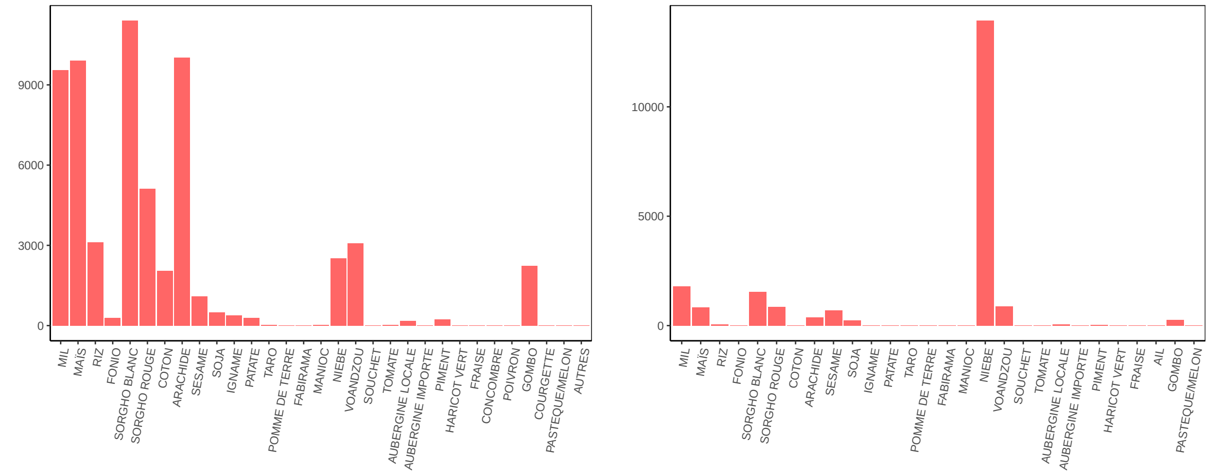

Figure 1: Crop choices for plot cultivations (above: main crop, lower: second crop, 2009 and 2011).

(Burkina Faso), ranging 2003 to 2017. The agricultural dataset is at plot level while many of the variables in the household survey are at individual level. The main estimations of the paper are done at a plot level, therefore we have chosen to maintain all the information at plot level and aggregate individual variables in the household-level dataset at a household level, to then merge together via unique household identifiers into a large plot-level dataset. Villages are then geolocalized by matching the survey datasets with administrative level data by fuzzy string matching in order to bypass inconsistencies in village names due to different dialectal inflections and spelling variations. Households are not identified geographically, and we are able to localize the data only at a village level. Overall the dataset contains 19,993 unique households located in 1,950 villages (see Fig.(1a) in the paper), cultivating 202,162 unique plots over the years. Each household on average cultivates around 10 plots throughout the years. Each plot can have up to two different crops, among 31 possible ones. The most common food crops are millet, corn, rice, sorghum and peanuts, and the only cash crop is cotton (traditional, bio, GMO). Figure 1 shows the counts of crop choices per yield in the 2009-2011 dataset. Most crops are food crops, and cash crops are almost entirely chosen as primary crop. As shown in Figure 14, the distribution of plot yield is identical after partialling out household fixed effects, implying heterogeneity is substantially generated at a plot level. Figure 2 shows how the villages cover substantially the area of Burkina Faso, and allow us to exploit a large variation in terms of both disease prevalence and geographical heterogeneity.

Table 1: Repeated households

	2011	2012	2013	2014	2015	2016	2017
2003	4	4	4	2	2	2	2
2005	5	6	6	3	3	3	3
2006	5	6	6	2	2	2	2
2007	5	6	6	3	3	3	3
2008	119	120	128	19	18	18	19
2009	3,040	2,994	3,185	10	10	10	11
2010	3,049	3,066	3,208	18	19	18	17

Tables 1 and 2 show which households and villages are repeated across the 2010 cutoff, and are roughly equivalent in the years between 2009 and 2013. For the estimations of the paper we choose 2009 and 2011 for

robustness, number of observed plots and model fit, although all results hold for other year combinations. Figure 2 presents the maps of the villages for the five relevant years around the 2010 cutoff (2009 to 2013) and their aggregated average yield.

Table 2: Repeated villages

	2011	2012	2013	2014	2015	2016	2017
2003	18	18	19	16	16	16	16
2005	25	25	26	23	23	23	23
2006	25	25	26	21	21	21	21
2007	26	26	27	23	23	23	23
2008	651	648	697	235	233	235	236
2009	617	616	657	133	131	133	134
2010	588	586	618	125	124	125	126

1.2. Climatic, environmental and malaria covariates

Table 3 gives details on the data sources used in our analysis, and yearly means of the climate covariates are shown in Figure 3.

1.3. Transforming schistosomiasis prevalence into infection intensity

The input to our analysis consists of modelled estimates of schistosomiasis prevalence, obtained via Bayesian geostatistical methods, over a pixel grid at 5×5 km. For all details of the first map we refer to the original paper[6], and the second is estimated in the same way. However, disease burden is more directly related to infection intensity (worms per person), rather than prevalence. We therefore proceed to transforming modelled prevalence into infection intensity using common assumptions about the distribution of worm burden in the human population.

The data consists of prevalence and mean intensity of infection from parasitological surveys in Burkina Faso coming from the Schistosomiasis control program of the Ministry of Health[7]. All samples correspond to school-aged children. We here make the assumption that the number of *Schistosoma* eggs per sample in the population follows a negative binomial distribution[8,9]. We have that the prevalence p is given by

$$p = 1 - P(X = 0) = 1 - \left(1 + \frac{\mu}{k}\right)^{-k}.$$

Table 3: Details on remote sensing climatic and environmental covariates

	Website	Source	Covariates
Daily rainfall	CHIRPS-V2	1	Yearly rainfall<br>Dry-period rainfall<br>Wet-period rainfall<br>Mean length of dry spells<br>Max length of dry spells
Monthly mean surface temperature	MODIS MOD11A1	2	Mean yearly surface temperature (day)<br>Mean yearly surface temperature (night)
Monthly mean air temperature		3	Mean yearly air temperature<br>Mean yearly night temperature<br>Mean dry-period day temperature<br>Days with temperature above seasonal median<br>Days with temperature below seasonal median
Vegetation indices	MODIS MOD13A2	4	Mean yearly Normalized Vegetation Index (NDVI)<br>Mean yearly Enhanced Vegetation Index (EVI)
Malaria	Malaria Atlas	5	Prevalence of *Plasmodium Falciparum* (2003-2017)

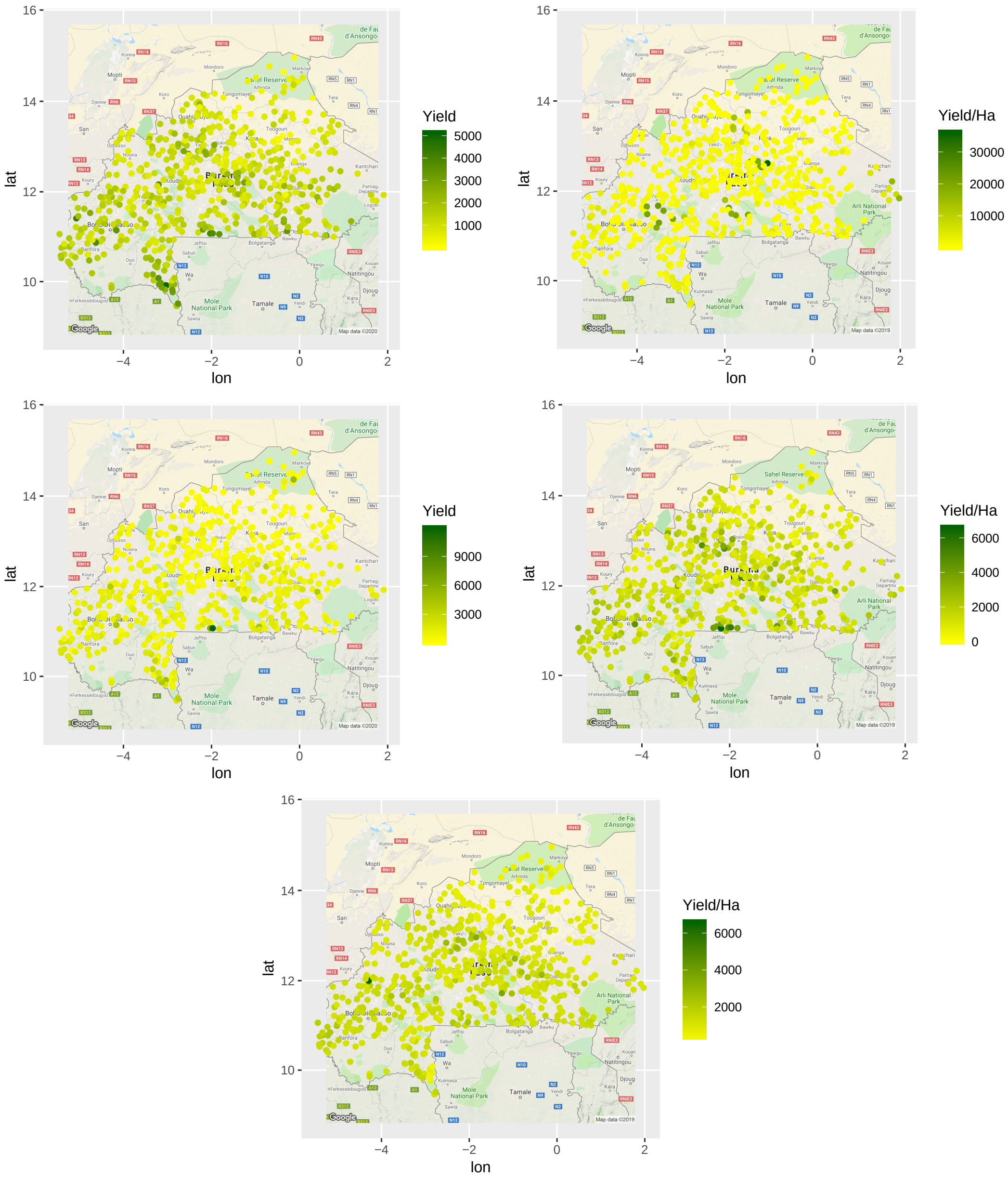

Figure 2: Geolocalized villages and relative average agricultural yield for the years 2009 to 2013 (from top left, in order)

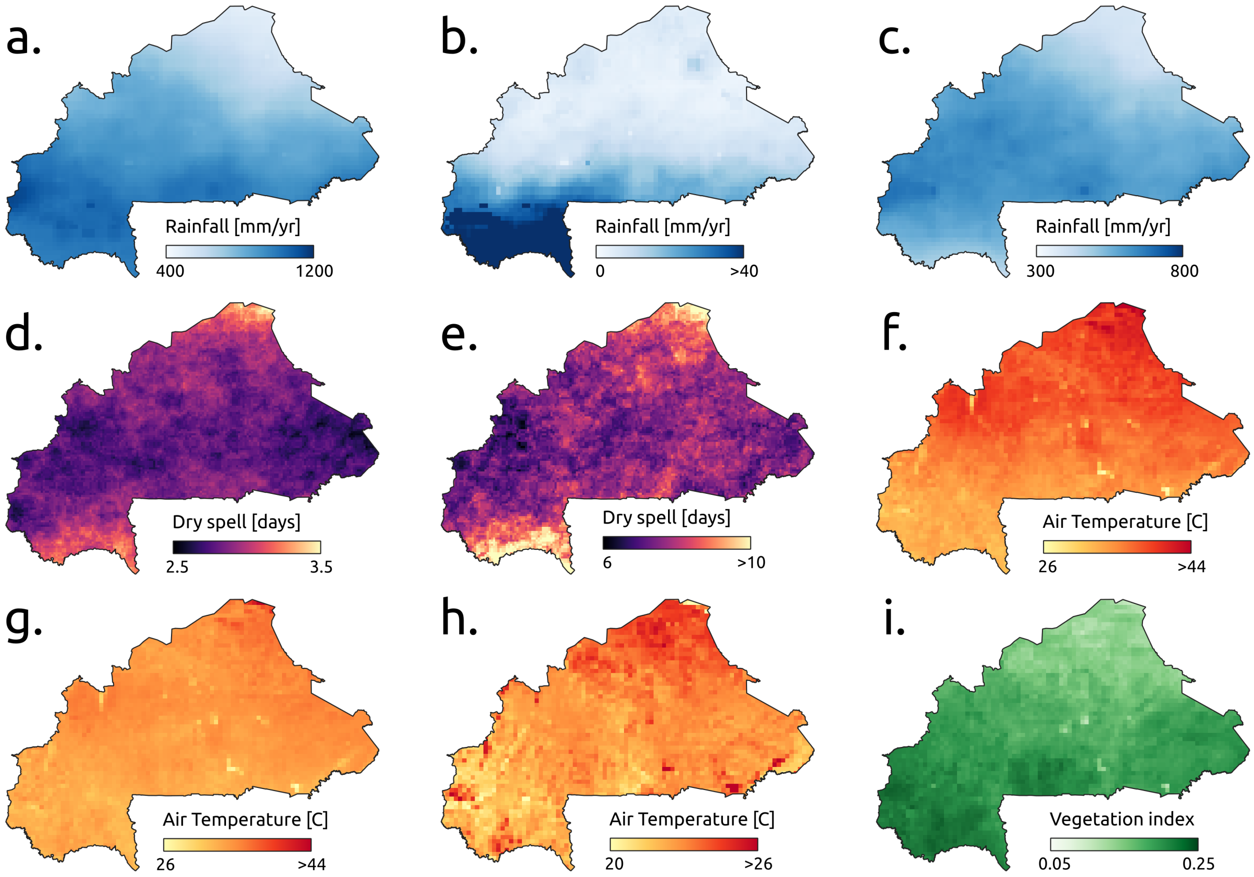

Figure 3: Climatic and environmental covariates used in the analysis. (a) Mean yearly precipitation, (b) mean dry-period precipitation (January to May), (c) Mean wet period precipitation (June to October), (d) Mean length of dry spells during the wet period. (e) Mean of maximum length of dry spells during the wet period, (f) Mean dry-period air temperature, (g) Mean day temperature, (h) Mean night temperature, (i) Mean Enhanced Vegetation Index (EVI). All figures show the yearly mean for the 2004-2018 period.

Parasitological data across endemic countries suggest that the aggregation parameter k is not constant across transmission settings, but rather varies as a function of the mean intensity of infection μ in the population (mean eggs/person)[9]. Following other studies, we test for either a constant, linear ($k(\mu) = a + b\mu$) or a quadratic ($k(\mu) = a+b\mu+c\mu^2$) relation. Inference on $k(\mu)$ is drawn through maximum likelihood estimation. For a given parameters set θ, the likelihood of the parameters the parasitological data is given by a binomial distribution on the number of infected people $n_{infected,i}$ among the sampled population $n_{sampled,i}$ in each village i among m sampled villages:

$$\mathcal{L}(\theta, \mathcal{D}) = P(\mathcal{D}|\theta) = \prod_{i}^{N} p_i^{n_{infected,i}} (1 - p_i)^{n_{sampled,i} - n_{infected,i}},$$

where p_i is the prevalence of schistosomiasis in village i. The negative log-likelihood is then minimized using standard optimization algorithms. We select for the best model using Akaike Information Criterion (AIC) AIC $= -2\log(\mathcal{L}) + 2m$, where m is the number of parameters in the model. We find the that there is a strong support for a non-constant functional form (Table 4). The best-fitting quadratic parameter is not statistically significant, so the linear form is retained.

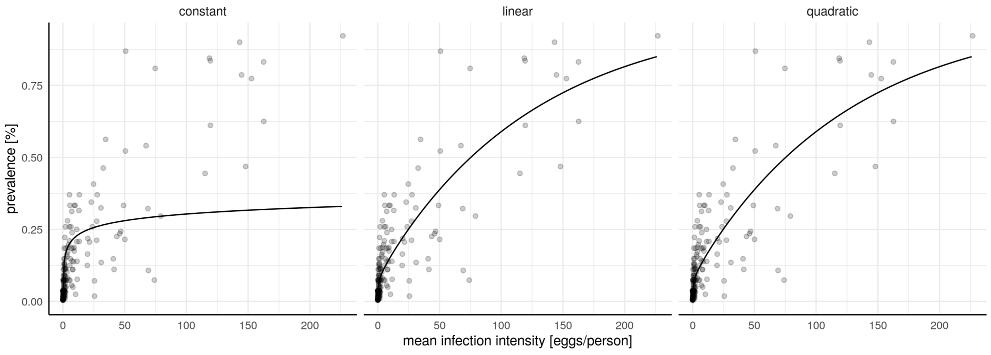

Figure 4: Functional forms of the variation of parasite aggregation with mean infection intensity in Burkina Faso. The best fitting relationship is a linear function of mean egg-intensity (Table 4).

Table 4: Model selection results for the functional form of the aggregation parameter for the inversion of schistosomiasis prevalence into infection intensity.

$k(\mu)$	$\log(\mathcal{L})$	AIC	parameters		
			a	b	c
constant	-6682.02	13366	0.047	-	-
linear	-6020.48	12043	0.016	0.0012	-
quadratic	-6020.48	12043	0.016	0.0012	1e-12

2. Methods

2.1. Framework and estimation methods

We now present in full detail the econometric framework and estimation methods used for each of the main three sections of the paper.

2.1.1. Schistosomiasis and agriculture

We begin by specifying the production technology as

$$Y_{ihjt} = A_{hj}(1 + \phi_{hjt})^{\theta} F(X_{ihjt}) e_{ihjt}, \tag{1}$$

where Y_{ihjt} is the yield (output per hectare) of plot i, farmed by household h in village j at time t, ϕ_{hjt}, which is common to all plots cultivated by a given household, represents the direct effect of schistosomiasis on productivity, calibrated by a parameter θ. We model the impact of the disease as $(1 + \phi)^{\theta}$ in order to represent the fact that at a disease-free equilibrium the household's production technology is unaffected, and decreases as the disease burden worsens. A_{jh} are household time-invariant productivity shifters, some of which may be unobservable, $F(.)$ is the production function and X_{ihjt} is the matrix of total inputs, including plot, household, climate and land covariates. Lastly, $e_{ihjt} = \exp(\alpha_t + \alpha_h + \alpha_c + \alpha_{ct} + \alpha_{ht} + \alpha_{ch} + \epsilon_{ihjt})$ represents plot-level unobservables, which we decompose into crop- (c), household- (h) and time-specific components (t) as well as their interactions, and the term ϵ_{ihjt} as an idiosyncratic Gaussian disturbance. The parameter of interest is θ: it modulates the extent to which the disease affects agricultural yield, and we expect its estimate to be negative. Since the "real" effect ϕ of the disease is unobservable, we proxy it with the measure of infection intensity in terms of mean egg-output per person discussed earlier, denoted by $I_{jt} = (1 + Int_{jt})$. Taking logarithms then results in the quasilinear model:

$$y_{ihjt} = \tilde{A}_{jh} - \theta I_{jt} + \tilde{F}(X_{ihjt}) + \alpha^{fe} + \epsilon_{ihjt}, \tag{2}$$

where $\alpha^{fe} = \{\alpha_t + \alpha_h + \alpha_c + \alpha_{ct} + \alpha_{ht} + \alpha_{ch}\}$ is the full set of fixed effects that account for all unobservables at a household, crop and year level as well as their interactions. Our goal is to identify the parameter θ. In all estimations, for the variables that include zeroes (particularly plot surface and yield) we use the inverse hyperbolic sine transformation instead of the natural logarithm. We begin by imposing a Cobb-Douglas functional form on $F(.)$, which yields a fully linear model. The instrumented model takes the same form as (2). Note, if the household is assumed to maximize profits over its set cultivated plots, that $\tilde{F}(X_{ihjt}, K_{ihjt})$ must be replaced by $\tilde{F}(X^*_{ihjt}, K_{ihjt})$, where $X^*_{ihjt}(A_{hj}, I^{-\theta}_{jt}, p_{jt}, K_{ihjt}, \epsilon_{ihjt})$ is input use at the optimum, p_{jt} represents input prices and K_{ihjt} represent all other factors of production not under the control of the household (for example, climate). In this case the total marginal effect of schistosomiasis on yield would be given by $-\theta + \sum_{w=1}^{w=W} \frac{\partial \tilde{F}(X^*_w, K)}{\partial X_w} \frac{dX^*_w}{dI}$, where W is the total number of inputs chosen optimally. In the next section we show that (1) is compatible with a model in which schistosomiasis affects *effective* labor input and that, empirically, $\frac{dX^*_w}{dI} = 0$ for all $w = 1 \ldots W$: schistosomiasis therefore is a pure productivity shock that does not affect optimal input use.

We then relax the restrictive assumptions (such as unitary elasticity of substitution between factors) imposed by the Cobb-Douglas functional form, and use adaptive machine learning methods in order to partial out all confounding and nonlinear effects of the factor inputs X_{ihjt}. The quasilinear structure of (2), stemming from the identification of schistosomiasis as a productivity shock, allows us to disentangle the effect of the disease from the effect of the other covariates. After obtaining a consistent estimate of the function $\tilde{F}(.)$, we can partial out its predicted values from both log-yield and schistosomiasis infection intensity, thereby identifying the effect of interest, θ. Since determining the precise parametric form of $\tilde{F}$ that best fits the data is of secondary interest in the present context, we use adaptive machine learning methods, which are obscure in terms of interpretation but well-suited for prediction and classification. This procedure is similar to the procedure named double/debiased machine learning (DML) [10–13]. The original procedure was proposed in order to deal with overfitting due to high-dimensional data and the estimation of treatment effects, and in this paper we extend it to the regularization of all confounding effects due to the unknown functional form for the production function from both outcome variable (yield) and disease intensity: this extension is one of the methodological contributions of the paper. The procedure needs to be cross-fit in order for it to be unbiased. Monte Carlo simulations (available upon request) of different production functions show how non-crossfit DML can actually increase bias. The method is readily extended to the case of multiple IVs: each of the IVs is partialled out, and the resulting residual used in a standard

IV estimation. The method requires imposing the double Neyman-orthogonal moment conditions:

$$\mathbb{E}[\psi(W,\theta,\eta_0)] = 0, \tag{3}$$

$$\partial_\eta \mathbb{E}[\psi(W,\theta,\eta)]_{\eta=\eta_0} = 0, \tag{4}$$

where $\psi(W,\theta,\eta_0) = ((y - \mathbb{E}[y|X]) - (I - \mathbb{E}[I|X])\theta)(I - \mathbb{E}[I|X])$ is the Neyman-orthogonal score function, $\eta_0 = (\mathbb{E}[y|X], \mathbb{E}[I|X])$, and where X is the complete matrix of covariates; ∂_η is a functional derivative operator, and the second condition imposes validity of the estimators under possible deviations from η_0. Fixed effects are partialled out beforehand. For the estimation of the unknown nuisance functions $\mathbb{E}[y|X]$ and $\mathbb{E}[I|X]$, we use random forests, gradient boosting machines and neural networks to obtain their predicted values and identify the parameter of interest θ, and we compare their performance using a mean-squared error criterion. Note that estimating $\mathbb{E}[y|X]$ is equivalent to estimating $\tilde{F}$. The machine learning estimators are all well-established in the literature[14]. This approach is easily extended to an IV procedure, under the maintained hypothesis that the exclusion restriction for the IVs hold, and adds one stage per instrument to the usual procedure in which the Neyman-orthogonal moment conditions are imposed on each of the IVs. Details on the machine learning architecture are the following: in general we use a 5-fold cross-validation for every method, and the best method is chosen by means of a mean squared error criterion, in the non-IV case is gradient boosting machines and for the IV case random forests. For random forests, we use 1000 trees, each with a minimum terminal node size of 5. Tuning is done by randomly sampling input variables for each split, with a step factor of 1.5 with improvement of 0.05, starting from the square root of the number of sampled columns. Gradient boosting machines are fit using a Gaussian distribution since in all cases both y and I are continuous, with 1000 trees each with a shrinkage parameter of 0.1, a within-fold cross-validation of 3 folds and 20% of the observations in the training set chosen to fit each subsequent tree. Neural networks work less well than the other two methods, although the estimates are not statistically different, but result in a greater MSE. They are fitted with 5 units per hidden layer with maximum allowable weights of 5000 with 1% decay and 1000 maximum iterations.

We also estimate a semi-parametric model in order to expose potential non-linearities in the effect of the disease, where θI_{jt} is replaced by the smooth function $\theta(I_{jt})$ in Eq (2). Here $\theta : \mathbb{R}^+ \to \mathbb{R}$ is estimated by minimizing the squared residuals with a smoothing penalty on the second derivative θ''. The smoothing parameter is chosen by cross-validation, and the degrees of freedom of the interpolating function are chosen iteratively by checking for zero signal on the residuals. The result is shown in the bottom panel of Figure 2 of the paper. The multidimensional splines are fit in a similar manner when studying the interaction of schistosomiasis intensity with other variables (malaria prevalence and distance to dams/rivers).

Aggregating at the village level, the quasilinear model for a representative household is of the form:

$$y_{jt} = \tilde{A}_j - \theta I_{jt} + \tilde{F}(X_{jt}) + \alpha_t + \epsilon_{jt}. \tag{5}$$

The model is then estimated by fixed effects and IV in the same manner as was the case at the plot-level. When controlling for spatial effects and spatial correlation, the model we fit after stacking observations and imposing a Cobb-Douglas functional form is the mixed spatial autoregressive model (SAR) given by:

$$y_{jt} = (1_{2j} - \rho W_j)^{-1}[I_{jt}\theta + \tilde{X}_{jt}\beta + \alpha_t + \epsilon_{jt}], \tag{6}$$

where W_j is the matrix of spatial weights given by the distance between the j villages, ρ is the spatial autoregressive parameter, and $\tilde{X}_{jt}$ is the matrix of factor inputs expressed in logarithmic form. We fit a model where both dependent and independent variables are spatially lagged. In order to check for the presence of spatial random effects, we fit a model with a random term $u(d)$ with mean zero and spatial covariance function $M_\nu(d)$. We adopt the standard choice of a Matérn covariance function, given by

$$C_\nu(d) = \sigma^2 \frac{2^{1-\nu}}{\Gamma(\nu)} \left(\sqrt{2\nu}\frac{d}{\rho}\right)^\nu K_\nu \left(\sqrt{2\nu}\frac{d}{\rho}\right).$$

where d is between-village distance, ν and ρ are positive parameters, K_v is the modified Bessel function of the second kind and Γ is the Gamma function. All parameters are then fit by maximum likelihood, allowing us to estimate the spatial correlation between village pairs, and to obtain its rate of decay as distance increases.

2.1.2. Schistosomiasis and poverty

The estimation of the reinforcing effects of poverty on the schistosomiasis burden requires first the identification of the households in a state of poverty. We examine the additional burden of the disease for households farming plots that belong to different quantiles of the joint distribution of plot surface and crop weight. Households in the lower reaches of this joint distribution correspond to those who are the most affected by poverty, almost entirely dependent on subsistence agriculture. We then interact schistosomiasis intensity with an indicator representing whether a given plot belongs to a specific quantile of the joint plot surface/crop weight distribution. The estimated equation is the following:

$$\begin{aligned} y_{ihjt} &= \tilde{A}_{jh} + \theta \tilde{I}_{jt} + \theta_{pj} \tilde{I}_{jt} \times \mathbb{1}_{(w_j, s_j) \leq Q_k^j} + \tilde{F}(X_{ihjt}) + \alpha_t + \alpha_h + \alpha_c + \epsilon_{ihjt} \\ Q_k^j &= \min\{w_{ihjt}, \mathrm{s}_{ihjt} : k \leq \Phi(w, \mathrm{surf}; t)\}, \end{aligned} \tag{7}$$

where Φ is the joint distribution of plot weight w and surface s for each year t. We estimate θ_{pj}, the coefficient associated with this interaction variable, while varying k on a grid ranging from the 20th to the 60th percentile of the joint distribution. The coefficients associated with these variables represent the added burden of schistosomiasis linked with the underlying plot characteristics, which are likely to be correlated with poverty at the household level.

In order to characterize this mechanism in terms of its link to household poverty, we then carry out a similar procedure where the indicator function is defined at the household rather than at the plot level. The interest is in the estimation of θ_{ph} in

$$\begin{aligned} y_{ihjt} &= \tilde{A}_{jh} + \theta \tilde{I}_{jt} + \theta_{ph} \tilde{I}_{jt} \times \mathbb{1}_{(w^h, s^h) \leq Q_k^h} + \tilde{F}(X_{ihjt}) + \alpha_t + \alpha_h + \alpha_c + \epsilon_{ihjt} \\ Q_k^h &= \min\left\{ \sum_{j=1}^{N_t^h} w_{ihjt}, \sum_{j=1}^{N_t^h} \mathrm{s}_{ihjt} : k < \Phi\left(w^h, \mathrm{s}^h; t\right) \right\}, \end{aligned} \tag{8}$$

where $\Phi\left(w^h, \mathrm{s}^h\right)$ is the joint distribution of total crop weight and plot surface farmed by each household and N_t^h is the number of plots farmed by each household each year. The cutoffs k in this case correspond to the lower 5% and 10% tails. Estimates in both specifications are from a log-linear model where schistosomiasis intensity is instrumented with a control function approach, and errors are cluster-bootstrapped at a village level.

2.1.3. Schistosomiasis and water resources development

For this set of results we aggregate the data up to the village level, and compute the geographical distance of each village from the closest dams and reservoirs. The interest lies in establishing whether the presence of a large dam affects the magnitude of the effect of schistosomiasis on agricultural yields, and thus to identify villages in provinces that directly benefit from the presence of the four main dams. Interacting the presence of a dam with our measure of disease intensity allows to disentangle the direct impact of dams, which should increase yields, from the deleterious indirect effects that they may produce by facilitating the diffusion of schistosomiasis. The explicit inclusion of the dams, however, generates spatial dependence in the data that does not vanish even when controlling for the highest possible level of unobservables (regions), and therefore for the estimation we rely on a spatial autoregressive specification (SAR) of the form

$$y_{jt} = (1_{2j} - \rho W_j)^{-1} [\theta_1 I_{jt} + \theta_2 \mathbb{1}_{dam} + \theta_{int} I_{jt} \times \mathbb{1}_{dam} + \tilde{X}_{jt} \beta + \alpha_t + \alpha_r + \epsilon_{jt}], \tag{9}$$

where $\alpha_{t,r}$ are time and region fixed effects, W_j is a matrix of spatial weights given by the between-villages distance in coordinate degrees and ρ is the spatial correlation parameter, fit by maximum likelihood. A visual representation of the network of village distances used as spatial weights is shown in the right panel of Figure 14. The panel also shows how spatial correlation decays quickly as geographical distance (in coordinate degrees) increases, when the presence of dams is not included in the analysis. This makes spatial analysis irrelevant for village-level regressions without the inclusion of the water resources variables, once the appropriate fixed effects are included (region, province or commune).

The previous results are then refined by accounting for each village's distance in km from the nearest dam or water reservoir. We estimate an adaptive spline for the interaction term $I_{jt} \times dist_j$, with time and region fixed effects. In order to disentangle the effect of large-scale infrastructures from the smaller ones, we interact the presence of a large dam with the distance from any water infrastructure as well as with the measure of intensity, and estimate the equation

$$\begin{aligned} y_{jt} &= (1_{2j} - \rho W_j)^{-1}[\theta_1 I_{jt} + \theta_2 dist_j + \theta_3 \mathbb{1}_{dam} + \theta_{3int} I_{jt} \times dist_j \times \mathbb{1}_{dam} + \theta_{2int1} I_{jt} \times dist_j + \\ & \quad \theta_{2int2} I_{jt} \times \mathbb{1}_{dam} + \theta_{2int3} dist_j \times \mathbb{1}_{dam} + \tilde{X}_{jt}\beta + \alpha_t + \alpha_r + \epsilon_{jt}]. \end{aligned} \tag{10}$$

where the main coefficients of interest are θ_{3int} and θ_{2int1}.

2.2. Schistosomiasis and optimal input allocation

We now present a simple model of optimal household input allocation with a distortion to labor input due to schistosomiasis. We posit the disease to be a shock to *effective* labor supply E, of the form

$$E = (\phi^{-\tilde{\theta}} L),$$

with $\theta > 0$, and that the total agricultural output of the household is generated by

$$Y = A(\phi^{-\tilde{\theta}} L)^{\alpha} F(X, K),$$

where A is total factor productivity, $\alpha < 1$ and $F(.)$ is the remainder of the production technology, dependent on k inputs$X$ chosen by the household (pesticides, livestock use) and inputs K which affect production but cannot be chosen (climate, land type). A household aims to maximize its instantaneous agricultural profits by choosing optimally$L$ and X, and is given by

$$\arg\max_{L,X} \Pi = p^y A(\phi^{-\tilde{\theta}} L)^{\alpha} F(X, K) - wL - \sum_{i=1}^{k} p_i X_i \tag{11}$$

where p_i are the input prices, w is the labor wage, and p^y is the price of output. The $k+1$ optimal allocations are then obtained by solving the system of $k+1$ first-order conditions:

$$L^*(A, \phi^{-\tilde{\theta}}, K, w, p), X_i^*(A, \phi^{-\tilde{\theta}}, K, w, p) \rightarrow \begin{cases} L^* = \left(\frac{\alpha p^y A F(X^*, K)}{w \phi^{\alpha\tilde{\theta}}}\right)^{\frac{1}{1-\alpha}} \\ X_i^* = F^{-1}\left(\frac{p_i \phi^{\alpha\tilde{\theta}}}{AL^*}\right) \end{cases}$$

where $p = (p^y, p_1, \dots, p_k)$ is the vector of prices. Assuming an interior maximum, depending on the properties of $F(.)$, the log-linearized optimal production is therefore given by

$$y^* = \tilde{A} - \theta\phi + \alpha L^*(A, \phi^{-\tilde{\theta}}, K, w, p) + \tilde{F}(X_i^*(A, \phi^{-\tilde{\theta}}, K, w, p), K),$$

which recovers the quasilinear structure of the equation (2) of the paper. If F is assumed Cobb-Douglas, then the model becomes fully linear.

The total marginal effect of schistosomasis on log-linearized optimal production, therefore, is given by

$$\frac{dy^*}{d\phi} = -\theta + \alpha \frac{dL^*}{d\phi} + \sum_{i=1}^{k} \frac{\partial F(X^*, K)}{\partial X_i^*} \frac{dX_i^*}{d\phi},$$

where $\theta = \alpha\tilde{\theta}$, and the specification becomes identical to Eq. (1) (i.e. schistosomiasis is a pure productivity shock) if $\frac{dL^*}{d\phi}$ and all the $\frac{dX_i^*}{d\phi}$ are zero. Note that this model can straightforwardly be extended to a more general form $F(L, X, K)$, but we choose this specification to better illustrate the choice of estimating Eq. (1) and choosing both log-linear and adaptive forms for F.

The condition of zero first derivatives is what we observe in the data: a first set of reduced-form evidence comes from studying the interactions of schistosomiasis on all input variables. We obtain a clear sign of zero signal: Table 5 reports the coefficients of the interactions, and omits both the rest of the controls as well as the interaction of schistosomiasis intensity on non-chosen inputs (climate, land type). Table 6 shows how for all of the choice input variables the marginal impact of schistosomiasis is not significant, hence validating our initial assumption of identifying schistosomiasis as a productivity shock in estimating Eq.(1). The results are robust to more flexible specifications (interactions, adaptive methods), as well as by instrumenting the intensity with the snail densities. All other controls used for each line of Table 6 are omitted. Unlike schistosomiasis, malaria does not seem to have a significant effect in this specification as a productivity shock, which we find to be a believable finding: malaria, by the nature of its health effects and burdens, is more consistent with a temporary shock to direct labor supply. This identification would have to be tested with a completely different strategy, which we leave to future research.

2.3. Snail densities as instrumental variables

The granularity of the schistosomiasis prevalence maps results in a disease measure that is constant at a village level. Estimating the mechanisms of interest, particularly the effect of schistosomiasis on agricultural yield and the characterization of the disease as a poverty trap, greatly benefits from the use of our rich dataset at a more disaggregated level, such as household or plot level. However, this is likely to suffer from a variety of endogeneity issues, especially related to error in measurement given that the "real" value of the disease burden is likely to vary within each village. We therefore address these issues with the use of snail densities as an IV. Our approach requires a detailed justification. The prevalence measure that we use in our analysis is a joint measure, generated by averaging the two forms (intestinal and uro-genital) and subtracting the covariance[6]. We therefore need to instrument the two forms of the disease by including information on the different species of snail hosts. The intestinal form caused by *S. mansoni* is concentrated in the southwest part of the country, and its range is constrained by the presence of its intermediate host snails, *Biomphalaria pfeifferi*[15]. This snail species is not present outside of this region due to its sensitivity to prolonged habitat dryouts which are more common in the Central and Northern parts of the country[15,16]. As an instrument for intestinal schistosomiasis we therefore use the mean of *Biomphalaria* abundance for the villages in the southwest. The uro-genital form of the disease, caused by a different species of schistosomes, *S. haematobium*, is spread more uniformly throughout the country due to the ubiquity of the snail species of the genus *Bulinus* which serve as intermediate hosts. The highest prevalence of uro-genital schistosomiasis is found in the northern part of the Sahel. As opposed to *Biomphalaria*, *Bulinus* are present in a wide range of natural and man-made habitats throughout the country[16], and we use estimates of its abundance in ponds and zones with ephemeral rivers in both the rainy and the dry season in order to capture changes in river size. We use gridded predictions of the seasonal variations of snail densities, and in order to avoid simultaneous dependance on unobservable we use the one-year lagged seasonal predictions. Relevance of the instruments is maintained since one of the main drivers of snail abundance is autocorrelation[3]. In our dataset, the correlation between disease intensity and lagged snail abundance is positive and strong, especially with *Biomphalaria* in rivers in the southwest, and with *Bulinus* in the northern dry regions where rivers are more ephemeral. In all estimations we used both a Hausman-Wu test and a control function approach in order to validate relevance and strength of our instruments. Table 7 shows the relevance of the

Table 5: No signal on schistosomiasis interactions for choice inputs

	y: Log Yield
intensity×log surface	−0.001 (0.003)
intensity×presence of hired labor	−0.005 (0.003)
intensity×presence of motorized labor	0.008(0.008)
intensity:×presence of manual labor	−0.012(0.011)
intensity×presence of labor from mutual aid	0.002 (0.003)
intensity×total agricultural livestock	0.001(0.001)
intensity×total livestock	0.00000(0.00004)
intensity×cows	0.0003 (0.0003)
intensity×horses	0.007(0.004)
intensity×pigs	−0.0001(0.0004)
intensity×goats	0.0001(0.0003)
intensity×self-consumptions	0.0001(0.0003)
intensity×cows sold	−0.001 (0.001)
intensity×cows bought	−0.001(0.001)
intensity×pigs sold	−0.00001(0.001)
intensity×pigs bought	0.001 (0.001)
intensity×goats sold	−0.0001(0.001)
intensity×goats bought	0.00005 (0.001)
intensity×gifts	−0.001(0.0005)
intensity×thefts	0.0001(0.0001)
intensity×type of plot farming	0.003 (0.002)
intensity×npk (kg)	0.00000(0.00003)
intensity×urea (kg)	−0.0001(0.0001)
intensity×phosphates (kg)	−0.0001(0.0002)
intensity×solid pesticides (kg)	−0.00001(0.00001)
intensity×liquid pesticides (cl)	0.00000(0.00000)
intensity×herbicide (g)	0.00000 (0.00000)
intensity×herbicide (cl)	0.00001 (0.00001)
intensity×fungicide (g)	−0.00004(0.00005)
intensity×fungicide (cl)	−0.00001 (0.00001)
intensity×rodenticide (g)	0.00000(0.00001)
intensity×rodenticide (cl)	0.00002(0.00002)
intensity×number of household members	0.0001(0.001)
intensity×average age	−0.0001 (0.0002)
intensity×number of families in the household	−0.0001(0.006)
intensity×number of children	0.001(0.001)
intensity×number of young working	−0.0003 (0.001)
intensity×number of women working	−0.0003(0.001)
Observations	51,055
R^2	0.336
Adjusted R^2	0.290
Residual Std. Error	1.209 (df = 48072)
Time, crop and household f.e., village clustering	*p<0.1; **p<0.05; ***p<0.01

Table 6: Input use and schistosomiasis intensity

y	*Intensity (Clustered s.e.)*
log plot surface	0.0003 (0.0002)
presence of hired labor	0.0003(0.0004)
presence of motorized labor	0.0008(0.0008)
presence of manual labor	0.0002(0.001)
presence of horse-driven labor	-0.001 (0.0007)
presence of labor from mutual aid	-0.002(0.001)
total agricultural livestock	0.0001(0.001)
total livestock	0.0001(0.0008)
cows	-0.0011(0.0021)
horses	-0.0024(0.0039)
pigs	-0.0014(0.0028)
goats	0.001(0.0021)
self-consumption	0.0018(0.0014)
cows sold	0.0002(0.0015)
cows bought	-0.0042(0.0028)
pigs sold	0.0004(0.002)
pigs bought	0.0008(7e-04)
goats sold	0.0002(5e-04)
goats bought	-0.0001(0.0012)
gifts	0.0001(0.001)
thefts	0.0011(0.001)
type of plot farming	-3e-04(0.0012)
npk (kg)	0.0023(0.0016)
urea (kg)	-0.0025(0.0015)
phosphates (kg)	0.0006(0.0006)
solid pesticides (kg)	0.0015 (0.001)
liquid pesticides (cl)	0.0014(0.0012)
herbicides (g)	0.0025(0.0036)
herbicides (cl)	-0.0014(0.0032)
fungicides (g)	-0.0017(0.0023)
fungicides (cl)	0.0008(0.0006)
rodenticides (g)	0.0036(0.0025)
rodenticides (cl)	0(0.0013)
number of household members	-0.011(0.0042)
number of families in the household	0.0006(0.0007)
number of young members working	0.0045(0.0026)
number of women working	0.0052 (0.0029)
rainfed agriculture	0.0001(0.0001)
Observations	51,055
Time, crop and household f.e., village clustering	*p<0.1; **p<0.05; ***p<0.01

instruments (the first stage), while Table 8 reports the significance of the residuals of the first stage in the structural equation, which implies rejection of the hypothesis of exogeneity.

Table 7: First Stage

	Dependent variable: Schistosomiasis Intensity
	intensity
Biomphalaria, rainy season	2.063***
	(0.205)
Biomphalaria, dry season	0.970**
	(0.423)
Bulinus	0.204***
	(0.077)
Observations	51,055
R^2	0.422
Adjusted R^2	0.420
Residual Std. Error	9.439 (df = 50894)
Note: Other covariates omitted. Errors clustered at village level	*p<0.1; **p<0.05; ***p<0.01

Table 8: Rejection of exogeneity. Control function approach

	Dependent variable: Log Yield
First Stage Residuals	0.012**
	(0.006)
Observations	51,055
R^2	0.180
Adjusted R^2	0.178
Residual Std. Error	1.302 (df = 50895)
Note: Other covariates omitted. Errors clustered at village level	*p<0.1; **p<0.05; ***p<0.01

3. Schistosomiasis and agriculture

3.1. Full results

We present results for households and villages observed in 2009 and 2011. These years are chosen to maximize the number of observations and provide the best fit and accompanying model diagnostics. Results are broadly similar for other combinations of years for which there are enough repeated observations.

Figure 5 reports the different estimates of θ. Given that the intensity measure is expressed in terms of worm eggs per person, and that its value is in the 0-110 range, the coefficient associated with intensity is expected to be negative and of the order of 10^{-2}-10^{-3}. The point estimate represents the marginal impact of one additional worm egg per person on log yield.

The upper three estimates in Figure 5 impose a Cobb-Douglas functional form, yielding a fully linear model; the first includes year and crop fixed effects, the second adds household ones. The point estimates

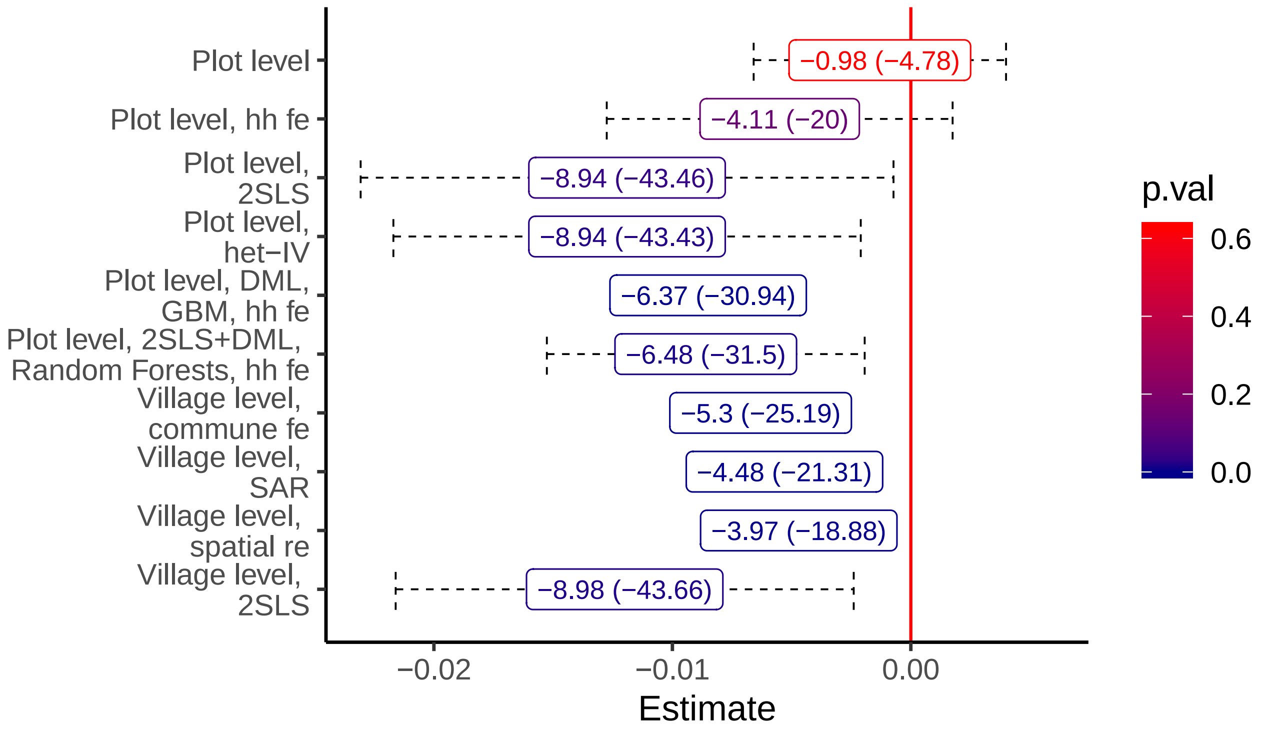

Figure 5: Estimates of the loss in yield due to schistosomiasis for 2009 and 2011 (95% confidence interval). Each label reports average and top 5% percentile losses. All estimations include the full set of controls and time fixed effects, and plot-level estimations also include crop fixed effects. Errors are clustered at village level (commune level for commune fe), and cluster-bootstrapped for 2SLS.

imply a loss in mean yield of between around 1% and 4%, with villages in the top 5% quantile of disease intensity losing between 5% and 20%; however, the coefficients are not statistically significant at usual levels of confidence. While time- and crop-invariant unobservables are controlled for, endogeneity and measurement error remain a concern. Since our measure of disease prevalence/intensity is model-based, the associated prediction error is included as a covariate, thereby hopefully reducing measurement error associated with the key right-hand variable. However, since our intensity measure is constant at the village level, and because individual households may display heterogeneous levels of infection within a same village, measurement error in all likelihood remains. As such, we then proceed to instrument disease intensity with the density of the freshwater snails that serve as intermediate hosts of schistosomes, using densities of both snail species *Biomphalaria* and *Bulinus* in different seasons (dry, rainy and winter) in order to instrument both forms of the disease as described in the paper. The third entry in Figure 5 shows how instrumenting intensity results in the point estimate of mean yield loss rising to 8.48% (40.99% in the top 5% intensity quantile). Estimation done via a control function approach yields identical estimates and also allows to test whether schistosomiasis intensity is actually endogenous, and this hypothesis is not rejected. It is then of interest to inquire what could the source of this endogeneity be, whether from the likely presence of measurement error or from a wider set of causes including omitted variable bias. We can get a clearer idea of the cause by using heteroscedasticity as an internal instrument for the mismeasured value of schistosomiasis intensity in the log-linear (Cobb-Douglas) model, a two-step GMM procedure first presented by [17]. The necessary heteroscedastic covariance restriction arises both by means of the nested structure of the data and by natural occurrence. The results are almost identical to the 2SLS estimates, as shown in the fourth line of Figure 5, and thus offers a possible explanation on the sources of the endogeneity bias in the standard panel regressions. Reliance on internal instruments, however, whilst controlling for coefficient biases, does not necessarily warrant causality in the effect, and therefore we rely on the instrumented specifications (log-linear and machine learning) as the main results of the paper.

Once we relax the functional form assumption on the production technology and adopt DML techniques, point estimates of the marginal effect of schistosomiasis intensity fall slightly, but precision improves. Indeed, as is apparent in Figure 5, all DML 95% confidence intervals are entirely contained within the confidence interval of the corresponding quasi-linear model, once household fixed effects are accounted for. For our 2009-2011 sample, our preferred non-instrumented model is given by gradient boosting machines, and results in a mean yield loss of 6.22% (30.05% in the top 5% intensity quantile). All three adaptive methods (random forests, gradient boosting machines and deep neural networks) perform similarly, and differences in MSE between the three are marginal. The IV DML estimates, which are computationally expensive in that they require at least 2-fold cross validation, yield slightly larger marginal effects, which are again estimated quite precisely. The instrumented DML estimate implies a mean loss of yield of 6.63% (32.04% in the top 5% intensity quantile). As expected, village-level estimates are more precise: within-cluster correlation is dampened and measurement error is reduced. Estimation of the linear model with commune fixed effects yields a loss in yield of 5.38% (25.57% in the top 5% intensity quantile), while the IV results yield a loss of 9.94% (47.26% in the top 5% intensity quantile). Fitting a spatial lag model with a weight matrix obtained from distances between villages yields a similar point estimate of 4.48%.[3] The map in the right-hand panel of Figure 14 provides a graphical illustration of the weight matrix. We then check for the presence of spatial random effects. Point estimates are slightly lower, with an average yield loss of 4%, but the null that this is equal to previous estimates cannot be rejected. We then construct a measure of spatial correlation from the parameters obtained from the spatial estimations. The right-hand panel of Figure 14 shows that spatial correlation decays rapidly: it vanishes almost completely at a distance of 1 degree (110 km at the equator), and by 0.33 degrees (ca. 37 km) it already falls below 0.1. This confirms the previous result that commune-level fixed effects already remove most spatial dependence in the data.

An interesting characteristic of our dataset is the homogeneity of households in terms of yield: in Figure 14 we plot the density of yields in levels as well as the corresponding density after the within-village trans-

[3]The choice of queen or rook-type adjacency weights does not significantly affect the estimates.

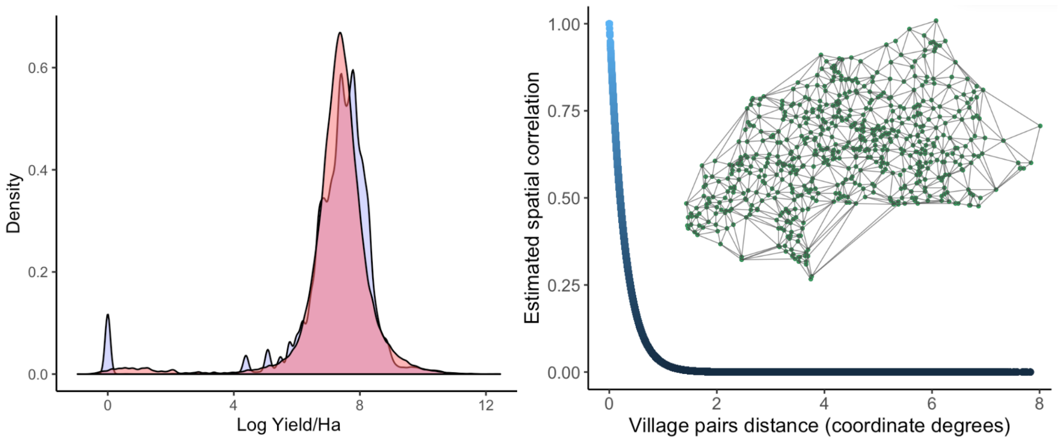

Figure 6: (Left) Homogeneity of households: original density of yield vs. rescaled density with village fixed effects partialled out. (Right) Distances between villages as spatial weights (inset), and the estimated spatial correlation.

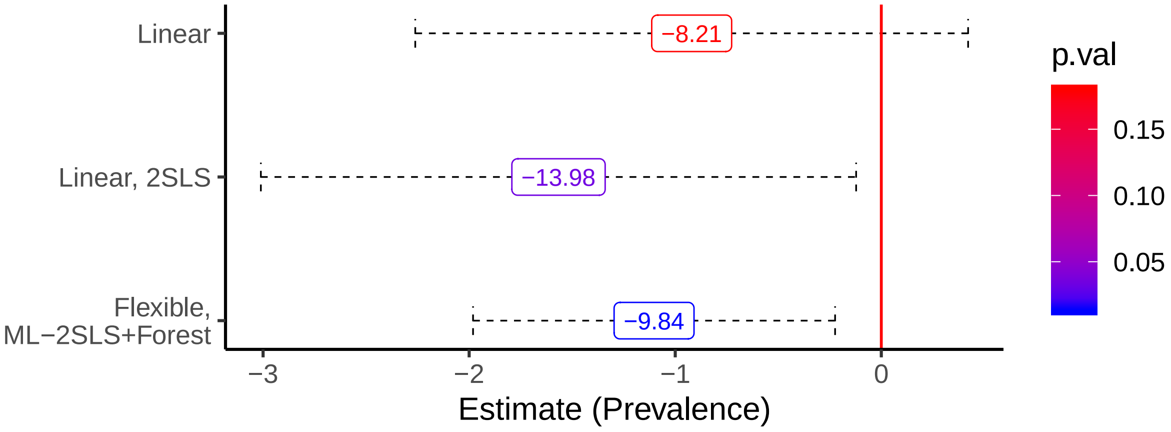

Figure 7: Estimates of the impact of schistosomiasis prevalence

formation. The densities are almost visually identical, and a Kolmogorov-Smirnov test cannot reject the null of equal distributions. Similar findings have been previously obtained [18], using Burkina Faso ICRISAT survey data from 1981-1983. Furthermore, the yield density remains equally unchanged after partialling out household unobservables: plot-level heterogeneity, therefore, seems to be driven by plot characteristics. We then aggregate our data up to the village level: given the aforementioned homogeneity of household yields, village aggregation leads to what is essentially a representative household model.

Figure 7 shows the robustness of the results to using directly the measure of schistosomiasis prevalence instead of intensity: the standard panel regression remain statistically insignificant, and the instrumented ones are not statistically different from the intensity ones but higher in terms of estimated percentage loss of yield (9.5% instead of 6.48%). The estimates reported in the main paper are therefore on the conservative side: the reasons behind this slight variation could lie in the direct dependence of the prevalence measure from the climate controls used in the geostatistical model estimation.

3.2. Non-linearity and interactions

As a robustness check, we fit a spline for the log-linear estimation in different combination of years, and Figure 8 shows how the results hold. There is no capping out effect as in the figure in the paper, because

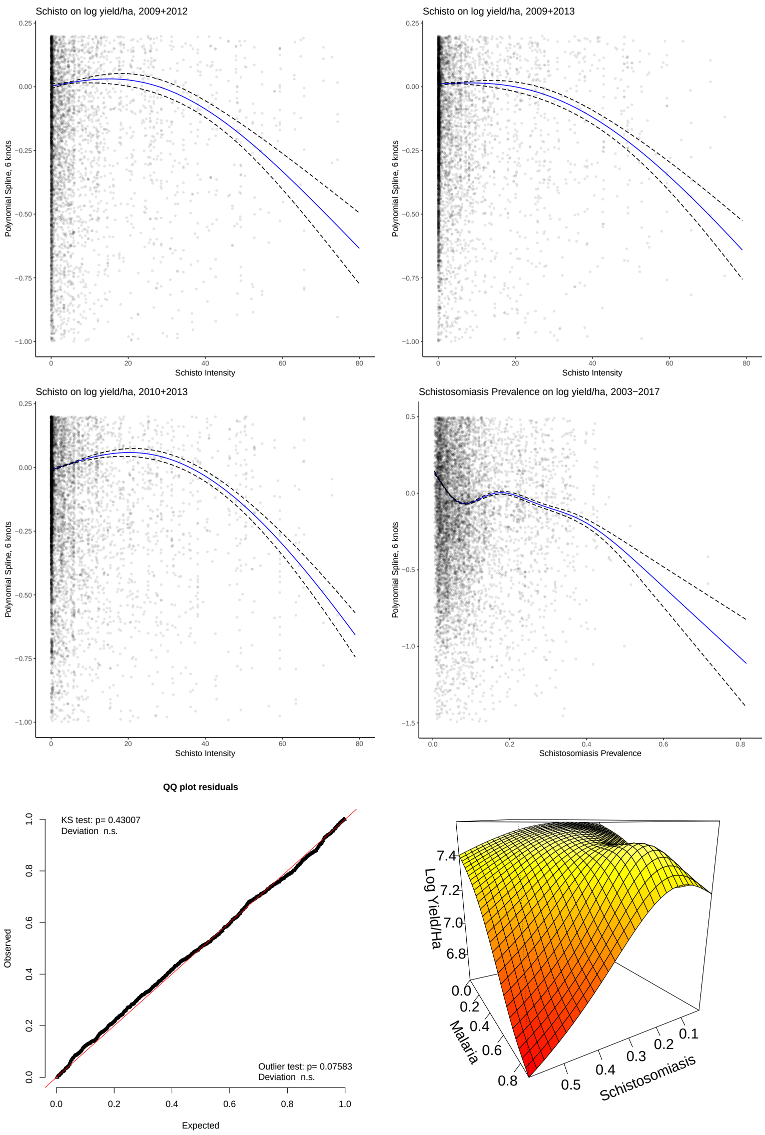

Figure 8: (Top) Spline fit for 2009, 2012 and 2013. (Middle) Spline fit for 2010, 2013, and using all years. (Bottom) Residuals fit for the 2009-2011 years (village level), and interaction of schistosomiasis and malaria prevalence measures.

the households in high-intensity areas (100+ worm eggs/person) that drive that effect are either trimmed or not surveyed in the above year combinations. We also fit a spline using all years (2003-2017) and the results stay consistent, although likely plagued with a variety of issues, since there are effectively only two data points per village in time. The lower left panel of Fig.8 shows the good residuals fit for the log-linear estimation of Figure 2 in the paper. There is a small *positive* effect of schistosomiasis on agricultural yield for low levels of intensity, with the significantly negative marginal effect beginning at an infection intensity of 30 eggs/person. This small positive marginal effect for low intensity stems from the interaction between schistosomiasis and malaria: given that malaria is only available as a prevalence measure, we fit a multidimensional spline for the interaction between malaria and schistosomiasis *prevalence*. The relationship is highly nonlinear, as displayed in the right hand panel of Figure 8: high prevalence levels for both diseases correlate with low crop yields, but low to intermediate levels of schistosomiasis mitigate the negative effects of high malaria prevalence. The immunological mechanisms behind this phenomenon have been previously discussed[19] and[20], presenting evidence on how low- to mid-intensity schistosomiasis can protect from acute malaria infections.

3.3. Other determinants of agriculture in the instrumented linear regressions

Table 9 reports the rest of the coefficients for the linear estimations. For clarity we only report the ones significant at standard levels of confidence: because of the nature of the data, constant at a village level, and the procedure being 2SLS, errors are cluster-bootstrapped at a village level, and therefore increase largely estimation errors and many controls result statistically insignificant. All continuous covariates are in logs. Signs are usually as expected, with an interesting negative quadratic relationship between plot surface and yield, suggesting decreasing returns to scale in plot size. Figure 9 shows nonlinear estimates of the correlation of schistosomiasis and the size of the cultivated plot. The negative quadratic relationship between yield and plot size remains established, and it seems that middle-sized plots are the ones that generate the higher yield.

4. Schistosomiasis and poverty: subsistence farming, crop choices and burden heterogeneity

We investigate whether schistosomiasis affects households differently based on whether they cultivate cash crops (cotton) or food crops (all the rest, mostly of subsistence). The question is of substantial policy relevance: if schistosomiasis exerts a lower burden for cash crops, then policies aimed at poverty reduction and diversification of the agricultural sector will carry along a beneficial effect on the reduction of the economic impact of schistosomiasis. We first note that plots that cultivate cash crops are substantially larger than the rest: Figure 10 shows the density of the log surface of plots used for cotton and the overall surface density, and shows how plots that cultivate cotton as a cash crop are substantially larger than the others. Larger farms in Burkina Faso involve richer households, especially when agriculture is not anymore of the subsistence variety. Cotton, furthermore, is drought-resistant and mostly rainfall-fed, therefore less in need of plots being around large water reservoirs or networks. We expect the effect to be reduced for these crops: we also expect the productivity shock to be less harmful for farms and households where constraints are less binding and agricultural yield is not as directly crucial for survival. The upper panel of Figure 11 estimate the main plot-level model with the same techniques as the main estimation. Errors are cluster-boostrapped. Instrumentation of the model increases the estimates by a large amount, because subsetting to cotton crops reduces greatly the variation in reservoir area of the snail hosts, as well as their estimated density: IV estimates are then large and extremely noisy. In any case, there does not seem to be a significant adverse effect of schistosomiasis on households farming cotton as cash crop, and the reason is likely to be twofold. The first reason is in cotton being a drought-resistant crop and thus requiring less irrigation networks: in the paper we show how this directly reduces the effective burden of the disease. The second reason lies in the fact that farms and households farming cash crops own substantially larger plots and are on average much richer; this can have multiple implications, from increased access to clean running water to better sanitary conditions. As the lower panel of Figure 11 shows, food crops, and particularly smaller plots mostly dedicated to farming of the subsistence variety, seem to suffer most of the disease

Table 9: Other determinants of agricultural yield

	Dependent variable: log yield
Log surface	0.466***
	(0.054)
Log surface^2	−0.140***
	(0.025)
Presence of hired labor	0.106***
	(0.033)
Total livestock used in agriculture	0.057***
	(0.022)
Female livestock used for breeding	−0.051**
	(0.024)
Cows	0.029*
	(0.015)
Sales of cows	−0.049*
	(0.026)
Npk (kg)	0.020**
	(0.008)
Urea (kg)	0.028***
	(0.009)
Solid pesticides (g)	0.027*
	(0.014)
Herbicides (g)	0.015*
	(0.008)
Plot near houses	0.604**
	(0.283)
Plot near bushes	0.577**
	(0.283)
Plot near encampment	0.587**
	(0.287)
Plot on flatland	0.185***
	(0.040)
Plot farmed with half-moon technique	−0.665***
	(0.203)
Dead/live hedges	0.169**
	(0.080)
Maximum level of education in household: Non-alphabetized	−0.123**
	(0.057)
Maximum level of education in household: Alphabetized	−0.057*
	(0.034)
Maximum level of education in household: Primary	−0.162**
	(0.073)
Loss due to flooding	−0.982***
	(0.122)
Loss due to fire	−0.549***
	(0.117)
Loss due to drought	−0.680***
	(0.044)
Loss after harvesting	−0.987***
	(0.156)
Mean temperature	−0.04*
	(2.597)
Temperature in dry season	0.03**
	(1.586)
Precipitation in dry season	0.721***
	(0.227)
Max dry spell	−0.307***
	(0.113)
Mean of land surface temperature (day)	−0.02**
	(1.088)
Mean of land surface temperature (night)	0.022***
	(0.915)
Enhanced Vegetation Index (level)	4.8**
	(2.014)
Note: Time+hh+crop fe. Cluster-bootstrapped (village)	*p<0.1; **p<0.05; ***p<0.01

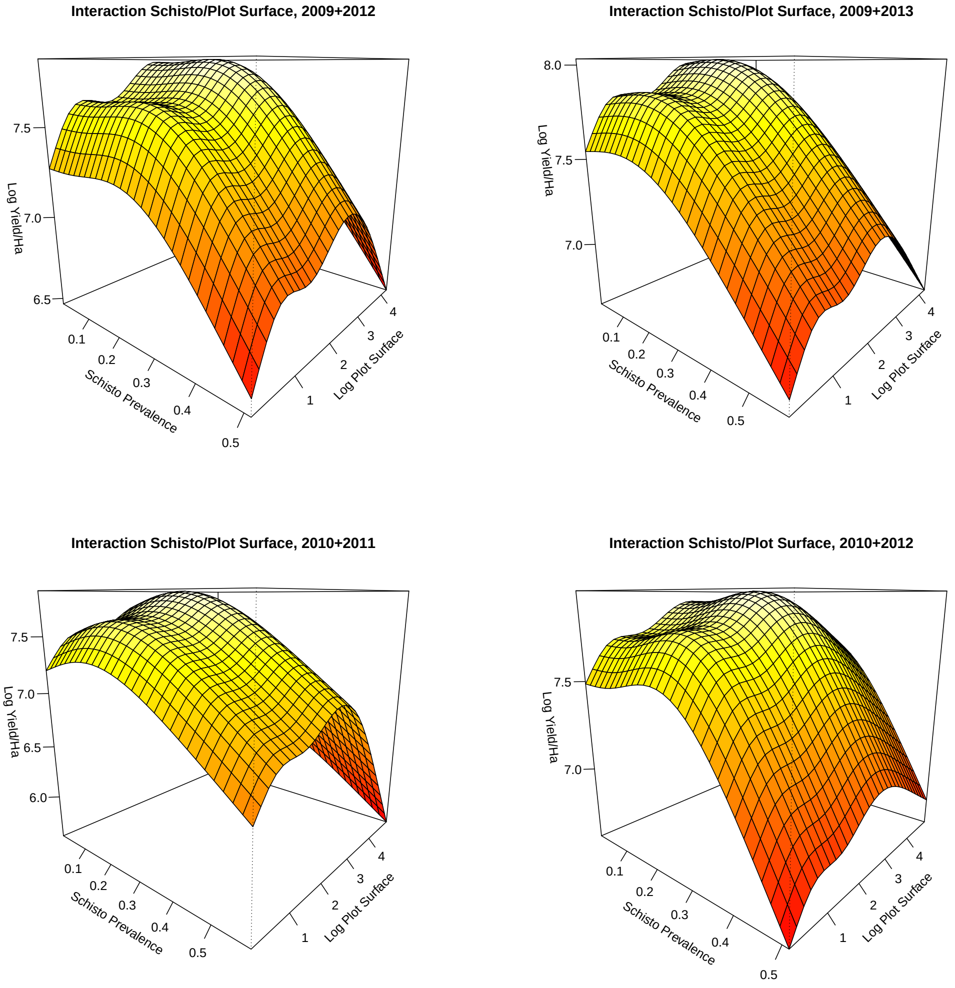

Figure 9: (Upper panel) Interaction of schistosomiasis intensity and plot surface (Other panels) Yield and plot surface for different levels of disease intensity

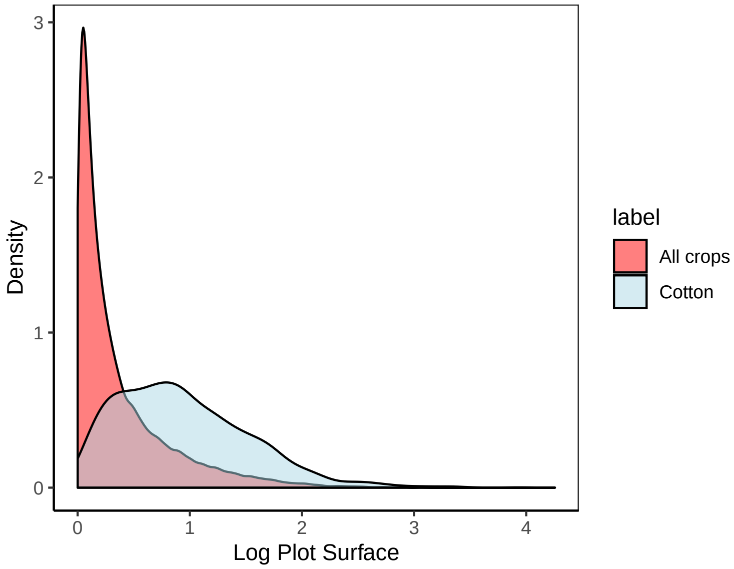

Figure 10: Distribution of plot sizes for cotton crops vs. all crops

burden, and hint at schistosomiasis being indeed a disease of poverty. Economic development aimed at raising individuals and households from having to farm for survival to a standard of agriculture with better returns and conditions will generate both an improvement in living standards and a reduction of the disease burden.

Table 10: Estimates of the interaction term for the poverty quantile and schistosomiasis intensity. Rows: surface, Columns: yield

	0.2	0.25	0.3	0.35	0.4	0.45	0.5	0.55	0.6	0.65	0.7
0.2	-0.015	-0.013	-0.011	-0.012	-0.013	-0.015	-0.015	-0.015	-0.014	-0.015	-0.014
0.25	-0.015	-0.013	-0.011	-0.012	-0.013	-0.014	-0.014	-0.013	-0.013	-0.013	-0.012
0.3	-0.013	-0.012	-0.011	-0.011	-0.011	-0.012	-0.011	-0.011	-0.010	-0.010	-0.010
0.35	-0.013	-0.012	-0.011	-0.010	-0.011	-0.011	-0.011	-0.010	-0.010	-0.010	-0.009
0.4	-0.012	-0.010	-0.009	-0.009	-0.009	-0.009	-0.009	-0.008	-0.008	-0.008	-0.007
0.45	-0.011	-0.010	-0.009	-0.008	-0.008	-0.009	-0.008	-0.007	-0.007	-0.007	-0.007
0.5	-0.010	-0.009	-0.008	-0.007	-0.007	-0.008	-0.007	-0.007	-0.006	-0.007	-0.006
0.55	-0.009	-0.008	-0.007	-0.006	-0.006	-0.007	-0.006	-0.006	-0.005	-0.006	-0.005
0.6	-0.007	-0.007	-0.005	-0.005	-0.005	-0.006	-0.005	-0.005	-0.005	-0.005	-0.005
0.65	-0.006	-0.006	-0.004	-0.004	-0.004	-0.005	-0.004	-0.004	-0.004	-0.004	-0.004
0.7	-0.005	-0.004	-0.003	-0.003	-0.003	-0.004	-0.003	-0.003	-0.003	-0.003	-0.003

Table 11: P-values for the interaction term for the poverty quantile and schistosomiasis intensity. Rows: surface, Columns: yield

	0.2	0.25	0.3	0.35	0.4	0.45	0.5	0.55	0.6	0.65	0.7
0.2	0.012	0.014	0.021	0.011	0.004	0.002	0.002	0.002	0.003	0.001	0.001
0.25	0.008	0.011	0.016	0.009	0.004	0.002	0.003	0.003	0.005	0.002	0.002
0.3	0.011	0.010	0.012	0.013	0.006	0.004	0.005	0.005	0.010	0.005	0.005
0.35	0.010	0.010	0.009	0.012	0.006	0.004	0.005	0.005	0.009	0.005	0.005
0.4	0.019	0.019	0.019	0.018	0.014	0.010	0.014	0.016	0.023	0.017	0.017
0.45	0.025	0.022	0.024	0.024	0.021	0.015	0.019	0.022	0.031	0.023	0.021
0.5	0.028	0.026	0.026	0.025	0.022	0.016	0.021	0.024	0.031	0.025	0.025
0.55	0.045	0.042	0.049	0.047	0.038	0.029	0.036	0.040	0.051	0.042	0.041
0.6	0.081	0.070	0.091	0.080	0.062	0.047	0.059	0.058	0.067	0.061	0.055
0.65	0.119	0.097	0.162	0.138	0.125	0.101	0.132	0.118	0.131	0.107	0.105
0.7	0.174	0.183	0.264	0.236	0.211	0.160	0.185	0.157	0.182	0.148	0.150

We now turn our attention to the heterogeneity of the disease burden for different joint quantiles of plot surface and plot yield. In the previous section we showed how cotton crops - cash crops - are on average much larger, and how for these crops the burden of schistosomiasis is small and not statistically significant. Cash crops are usually farmed by households which do not rely on subsistence agriculture, own larger plots

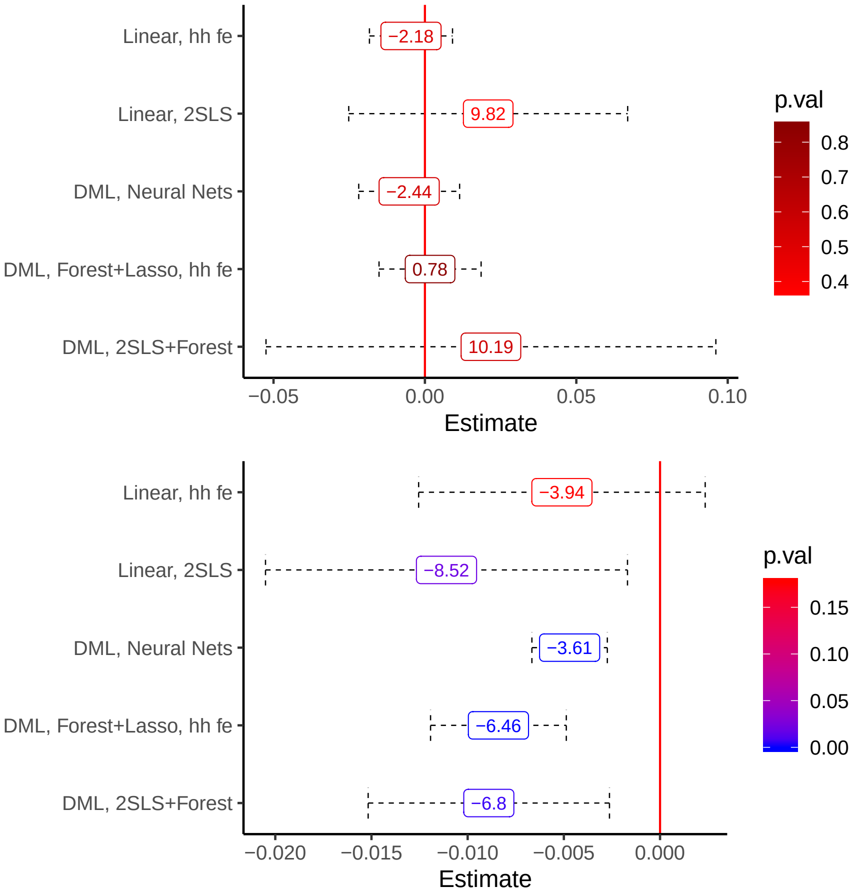

Figure 11: (Upper panel) Schistosomiasis burden for cash crops-only plots: no effect (Lower panel) Burden for food crops, mostly of subsistence: large effect. In labels: percentage yield loss for villages at the average schistosomiasis intensity.

Table 12: Coefficients of the interaction term for household at the bottom quantiles of total yield and plot surface (***: significant at 1%.).

	lower 5% surface	lower 10% surface
lower 5% yield	-0.044***	-0.039***
lower 10% yield	-0.049***	-0.045***

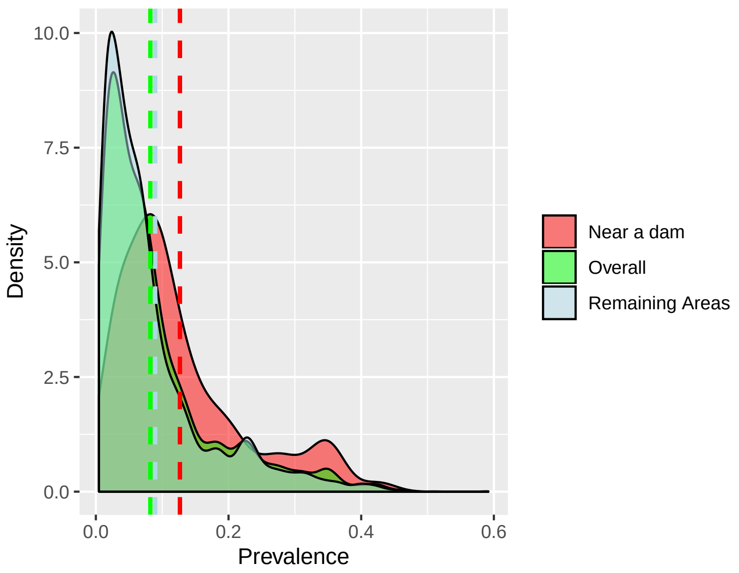

Figure 12: Distribution of schistosomiasis prevalence in areas near a large dam, plotted against the overall distribution and the distribution of the disease in the rest of the country.

and are thus on average richer. As stated in the paper, this is a first sign of how schistosomiasis causes and reinforces poverty, but it might be that smaller, poorer households also use cash crops (particularly GMO-resistent varieties of cotton) in order to reduce the risk of adverse shocks. This is not a common occurrence in our dataset, as seen in the histograms of Fig. 1. Table 10 shows the coefficients of the interaction term θ_{pj}, and Table 11 reports the respective p-values. These coefficients represent the *added* effect of schistosomiasis for the plots that are at the tail of the joint surface-weight distribution, for different quantiles. We note that the effect stops being significant around the 55th percentile. We then focus on households farming only food crops that lie at the very bottom of the joint distribution *between households*, grouping total harvest weight and overall plots surface per household and creating an indicator for the households at the 5th and 10th bottom percentiles. These households exhibit the lowest overall production and farm smallest plots, and thus belong to a situation of extreme poverty, especially given the overall state of agriculture in 2009-2011 Burkina Faso. Table 12 shows the coefficients θ_{ph} for the respective quantiles, the diagonal of which is represented in the paper. All estimates are from a log-linear model where schistosomiasis intensity is instrumented with a control function approach (standard 2SLS yields equivalent results), and errors are cluster-bootstrapped at a village level.

5. Schistosomiasis and water resources development: full results

We report here the full set of results for the analysis of schistosomiasis and water resources development. We focus on Burkina Faso's four main dams:the Bagré Dam in Boulgou province, which also involves Zoundwéogo, Kouritenga and Ganzourgou provinces, the Kompienga Dam in Kompienga province, the Ziga Dam in Oubritenga province and the Léry Dam in Nayala province. First we notice how the distribution of the schistosomiasis prevalence seems substantially higher in areas near the large dams, as shown in Figure 12. An interesting characteristic of our dataset is the homogeneity of households in terms of yield: Figure 14 shows the density of yields in levels as well as the corresponding density after the within-village transformation. The densities are almost visually identical, and a Kolmogorov-Smirnov test cannot reject the null of equal distributions. Furthermore, the yield density remains equally unchanged after partialling out household unobservables: plot-level heterogeneity, therefore, seems to be driven by plot characteristics.

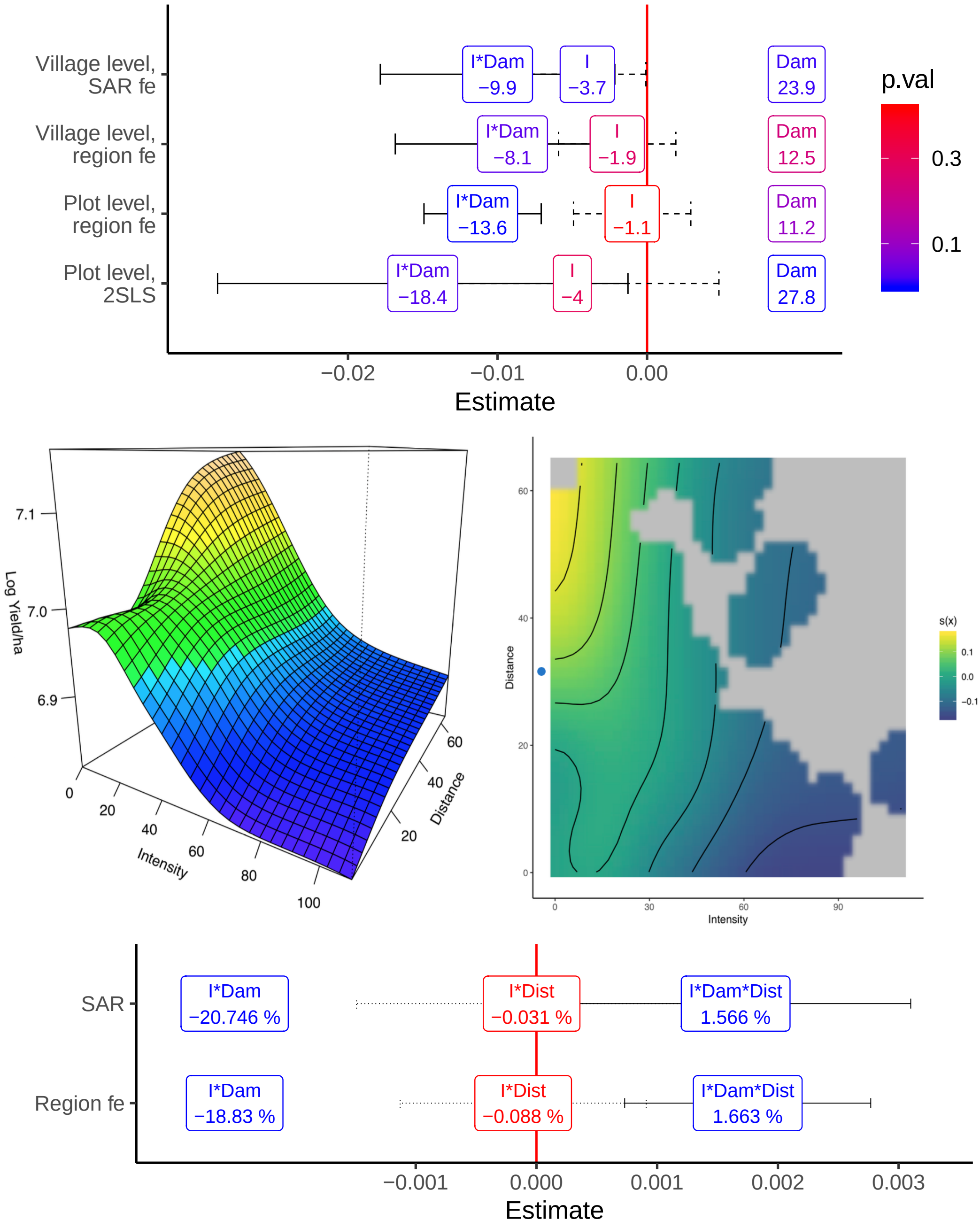

Figure 13: (Upper panel) Differential effect of schistosomiasis on yields caused by the presence of a large dam. (Middle panels) Estimated joint impact of schistosomiasis intensity and distance from all dams and water reservoirs. (Lower panel) Joint effect of schistosomiasis and distance from a large dam. All estimates control for time fixed effects, and plot-level estimates control for crop fixed effects. Errors are clustered at the regional level for region fixed effects estimates, and at the village level otherwise. 2SLS standard errors are cluster-bootstrapped.

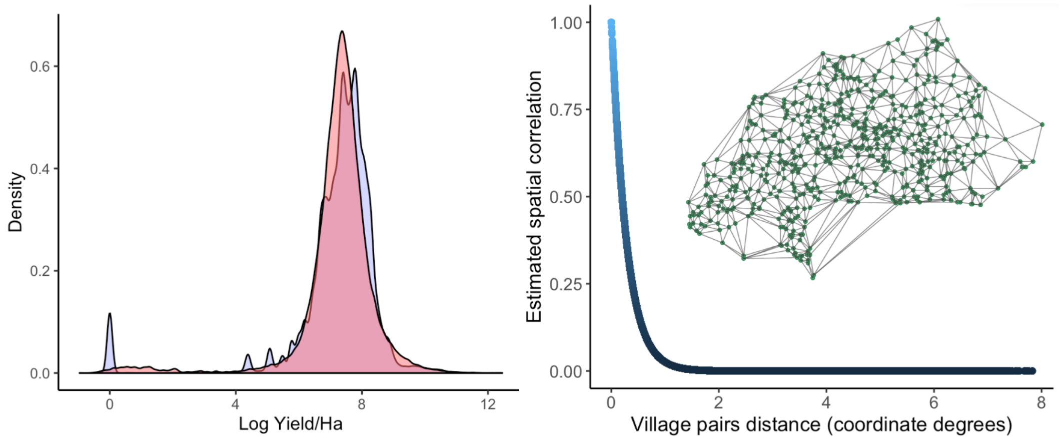

Figure 14: (Left panel) Homogeneity of households: original density of yield vs. rescaled density with village fixed effects partialled out. (Right panel) Distances between villages as spatial weights (inset), and the estimated spatial correlation.

Given the aforementioned homogeneity of household yields, village aggregation leads to what is essentially a representative household model.

We are interested in whether the presence of a large dam affects the magnitude of the negative impact of schistosomiasis on agricultural yields. The coefficient θ_{int} in (9) represents the difference in the effect of schistosomiasis on log yield attributable to the presence of a dam. We note that we cannot have household fixed effects, as this estimation would not allow to estimate the coefficient of the (non-interacted) dam variable, and we rely on region fixed effects instead. The upper panel of Figure 13 reports the results: irrespective of estimation method, the interaction term is always statistically significant at conventional levels of confidence, and the presence of a large dam increases the average loss of agricultural yield due to schistosomiasis by a minimum of 8.2% to a maximum of 18.7%. Plot level estimation yields mixed results: the interaction term remains negative and precisely estimated, while the intensity variable becomes statistically indistinguishable from zero whether one controls for region fixed effects or applies the 2SLS procedure.

We refine the previous results by accounting for each village's distance (in km) from the nearest dam or water reservoir. We start by fitting an adaptive spline to the interaction of each village's distance from the dams and schistosomiasis intensity. The full network of dams and reservoirs is illustrated in Panel (b) of Figure 1 in the main paper. Results are displayed in the middle panels of Figure 13: the left-hand panel shows how the deleterious marginal effect of schistosomiasis intensity on log yield is mitigated as one moves further away from a dam or a reservoir. The right-hand panel, based on the same estimation procedure, highlights how areas with high schistosomiasis intensity are concentrated within 20 km of a dam or reservoir. Households located in these areas suffer from large negative feedback effects between schistosomiasis and water resources development; to make matters worse, the effect of an increase in distance on the marginal effect of schistosomiasis intensity is greater for villages which display lower disease intensity. The bottom panel of 13 shows how the SAR framework and the region fixed effects one yield equivalent results. In order to find out what is the role of large dams in this mechanism, we include the triple interaction $I_{jt} \times \mathbb{1}_{dam} \times dist_j$, as well as all the double interactions, at a village level by both region fixed effects and SAR: the results are presented in the lower panel of Figure 13 and are very similar. The coefficient of the triple interaction is positive and significant: it has to be interpreted as the average amount to which the disease burden *decreases* as a village near any of the large dams moves one kilometer away from it. This implies an average *reduction* of around 1.5% (for SAR estimates) of the schistosomiasis burden on agricultural yield, starting from the average 18-20% loss suffered by the closest villages: being further away from large dams is therefore beneficial. The coefficient associated with the interaction $I_{jt} \times dist_j$ is always extremely small and insignificant at any reasonable level of confidence.

References

[1] C. Funk, *et al.*, *Scientific data* **2**, 1 (2015).
[2] Z. Wan, S. Hook, G. Hulley, Mod11a1 modis/terra land surface temperature/emissivity daily l3 global 1km sin grid v006 [data set]., NASA EOSDIS Land Processes DAAC (2015). Accessed 2020-03-06 from https://doi.org/10.5067/MODIS/MOD11A1.006.
[3] J. Perez-Saez, T. Mande, A. Rinaldo, *Geospatial Health* **14** (2019).
[4] K. Didan, Mod13a2 modis/terra vegetation indices 16-day l3 global 1km sin grid v006 [data set]., NASA EOSDIS Land Processes DAAC (2015). Accessed 2020-03-06 from https://doi.org/10.5067/MODIS/MOD13A2.006.
[5] K. E. Battle, *et al.*, *Lancet* **394**, 332 (2019).
[6] Y.-S. Lai, *et al.*, *Lancet Infectious Diseases* **15**, 927 (2015).
[7] H. Ouedraogo, *et al.*, *Bulletin of the World Health Organization* **94**, 37 (2016).
[8] R. Anderson, G. Medley, *Parasitology* **90**, 629 (1985).
[9] S. Brooker, S. Whawell, N. B. Kabatereine, A. Fenwick, R. M. Anderson, *Trends in parasitology* **20**, 537 (2004).
[10] A. Belloni, V. Chernozhukov, *Bernoulli* **19**, 521 (2013).
[11] A. Belloni, V. Chernozhukov, C. Hansen, *The Review of Economic Studies* **81**, 608 (2014).
[12] A. Belloni, V. Chernozhukov, I. Fernández-Val, C. Hansen, *Econometrica* **85**, 233 (2017).
[13] V. Chernozhukov, *et al.*, *The Econometrics Journal* **21**, C1 (2018).
[14] T. Hastie, R. Tibshirani, J. Friedman, *et al.*, The elements of statistical learning (2001).
[15] J. Perez-Saez, T. Mande, J. Larsen, N. Ceperley, A. Rinaldo, *Advances in Water Resources* **110**, 263 (2017).
[16] J. Poda, A. Traoré, B. K. Sondo, *Societé de Pathologie Exotique* **97**, 47 (2004).
[17] A. Lewbel, *Journal of Business & Economic Statistics* **30**, 67 (2012).
[18] C. Udry, *Journal of Political Economy* **104**, 1010 (1996).
[19] F. C. Hartgers, M. Yazdanbakhsh, *Parasite Immunology* **28**, 497 (2006).
[20] A. Degarege, *et al.*, *PLOS Neglected Tropical Diseases* **10**, 1 (2016).
